# Role of MicroRNAs in Protective Effects of Forsythoside A Against Lipopolysaccharide-Induced Inflammation in Bovine Endometrial Stromal Cells

**DOI:** 10.3389/fvets.2021.642913

**Published:** 2021-02-24

**Authors:** Haimiao Lv, Chenbo Yan, Lixin Deng, Zhan Peng, Dexin Yang, Wenjv Hu, Xuefen Ding, Chao Tong, Xinzhuang Wang

**Affiliations:** ^1^College of Veterinary Medicine, Henan Agricultural University, Zhengzhou, China; ^2^College of Agricultural Medicine, Henan Radio and Television University, Zhengzhou, China; ^3^Wushu Overseas Students Pioneer Park, Wuhu, China

**Keywords:** forsythoside A, microRNA, lipopolysaccharide, bovine endometrial stromal cells, endometritis

## Abstract

Bovine endometrial stromal cells (bESCs) are exposed to a complex environment of bacteria and viruses due to the rupture of epithelial cells after delivery. Inflammatory responses are elicited by the activation of host pattern recognition receptors through pathogen-related molecules such as lipopolysaccharides (LPS) on the cell membrane. Forsythoside A (FTA) is a major active constituent of *Forsythia suspensa* (Thunb.) Vahl. is a flowering plant widely employed as a traditional Chinese herbal medicine to treat various inflammatory diseases such as nephritis, eye swelling, scabies, ulcers, and mastitis; however, the molecular mechanisms underlying its therapeutic effects on bovine endometritis are still unclear. The aim of this study was to explore the role of miRNA and the mechanisms underlying the protective activity of FTA on the inflammation of bovine endometrial stromal cells induced by LPS. Based on previous research, we isolated and cultured bESCs *in vitro* and categorized them into LPS and LPS+FTA groups with three replicates. Upon reaching 80% confluence, the bESCs were treated with 0.5 μg/mL of LPS or 0.5 μg/mL of LPS + 100 μg/mL of FTA. We, then, performed high-throughput sequencing (RNA-Seq) to investigate the effects of FTA on LPS-stimulated primary bESCs and their underlying mechanisms. We identified 167 miRNAs differentially expressed in the LPS groups; 72 miRNAs were up-regulated, and 95 were down-regulated. Gene ontology enrichment analysis revealed that differentially expressed microRNA (DEGs) were most enriched during the cellular metabolic process; they were mostly located intracellularly and participated in protein, enzyme, and ion binding. Kyoto Encyclopedia of Genes and Genomes pathway analysis revealed that the DEGs were most enriched in the mitogen-activated protein kinase, tumor necrosis factor, and Interleukin-17 signaling pathways. These results reveal the complex molecular mechanism involved in the FTA and provide a basis for future studies of bovine endometritis treatment with traditional Chinese medicine monomer.

## Introduction

Uterine disease in dairy cows does not only have negative health consequences, such as infertility or reduced fertility but also leads to a major economic impact. Endometritis occurs due to vaginal and cervical dilatation and trauma, which causes bacteria in the vagina, skin, blood, feces, and environment to contaminate the reproductive tract (1). *Escherichia coli* is a common pathogen responsible for uterine infection in postpartum dairy cows. Lipopolysaccharide (LPS), which is located in the outer membrane of *E. coli* and other gram-negative bacteria, is a potent bacterial virulence factor that causes endometrial inflammation (2, 3). Toll-like receptors are one of the major families of pattern recognition receptors in innate immune cells that generate inflammatory responses by recognizing pathogen-associated molecular patterns, including bacterial DNA, lipopeptides, flagellin, and LPS (4). Bovine endometrial epithelial cells (bEECs) and bovine endometrial stromal cells (bESCs) encounter invading bacteria; thus, the endometrium represents the preliminary line of defense against invading bacteria. In fact, bEECs and bESCs also produce an inflammatory response to bacteria, lipopeptides, and LPS (5, 6). Previous reports have shown that in response to LPS, epithelial and stromal cells secrete cytokines via TLR4/MYD88-dependent cell signaling pathways (5). bEECs are essential because they form the first cellular barrier to infection in the intact endometrium; however, during parturition and in the postpartum period, the epithelium is disrupted and sloughed; thus, bESCs are also exposed to invading bacteria (7, 8). Besides, bESCs are far more abundant than bEECs, which are adjacent to the vascular system; therefore, the cytokines they produce may be more influential than the inflammatory mediators produced by epithelial cells (7–9). In this study, we used LPS-stimulated bESCs as an *in vitro* inflammatory model to explore a new method for treating endometritis.

MicroRNAs (miRNAs) are an abundant class of short, small, non-coding RNAs that are 21–23 nucleotides in length found in eukaryotic cells; they post-transcriptionally modulate the expression of their target genes by binding to the 3′-untranslated regions of messenger RNAs (mRNAs) (10, 11). MiRNAs play critical roles in the regulation of anti-inflammatory reactions. In this context, miR-135a restrains NF-κB activation, probably by targeting TLR4 to alleviate the silica-induced inflammatory response and pulmonary fibrosis (12). In a model of acute lung injury, the inflammatory response induced by LPS was attenuated by miRNA-223 through the NLRP3 inflammasome and TLR4/NF-κB signaling pathway via RHOB (13). Moreover, Schisandrin A attenuates LPS-induced damage in human HaCaT keratinocytes through microRNA-127-dependent regulation (14).

**Figure 1:**
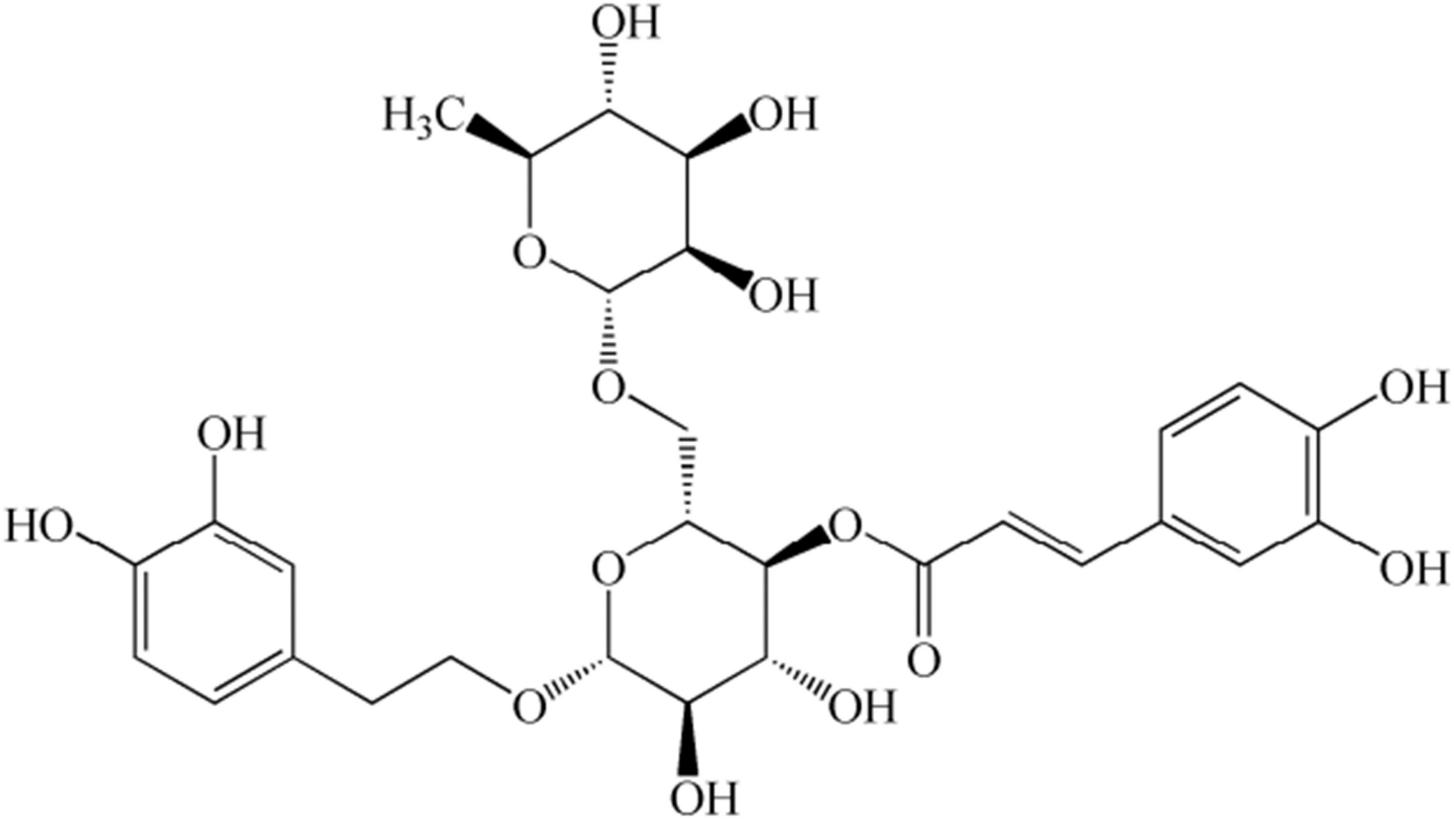
Chemical structure of forsythoside A (FTA).

For endometritis treatment, traditional Chinese medicine can not only achieve an ideal therapeutic effect but can also overcome the drawbacks of drug-resistant strains caused by antibiotics and hormone residues. *Forsythia suspense* (Thunb.) Vahl. is a flowering plant that is used as traditional Chinese medicine. Its earliest record is in *Shennong Bencao Jing*, a book on traditional herbal medicine (15). Clinical studies have shown that *F. suspensa* is often used to treat various inflammation-related diseases such as nephritis, eye swelling, scabies, ulcers, and mastitis (16). Forsythoside, which is one of the primary active components of *F. suspensa*, mainly persists as forsythoside A to J; among these, forsythoside A (FTA) (Figure 1) has been reported to exert a protective effect on inflammatory models (17, 18). In addition, FTA elicits a protective effect against LPS/GalN-induced liver injuries in mice (19). Furthermore, it has been demonstrated to elicit significant anti-endotoxin activity in LPS-treated mice and LPS-stimulated cells by blocking the LPS/TLR4 signaling pathway and inhibiting Tregs (20). However, the protective mechanism of FTA on LPS-induced inflammatory responses in bESCs is poorly understood. Therefore, this study aimed to explore the role and mechanism of miRNAs in the protective effect of FTA on the inflammation of bESCs induced by LPS.

## Methods

### Bovine Endometrial Stromal Cell Isolation and Culture

Fresh bovine uteri were collected from a local abattoir and maintained on ice until further processing in the laboratory. The health of the bovine was objectively confirmed through the veterinarian's examination of government and individual records of bovine well-being. There was no purulent or mucopurulent vaginal secretion, and there were no signs of clinical or subclinical endometritis. In addition, the uterus sample showed no signs of genital disease or microbial contamination, and there were no ovarian cysts. All experimental procedures were approved by the Institutional Animal Use Committee of Henan Agricultural University (approval number 2005-0026) and the Beijing Association for Science and Technology (approval SYXK [Beijing] 2007-0023).

Separation and culture of bESCs were performed as previously described (21), with minor modifications (22). Briefly, under sterile conditions, the tissue mass was cut into 1-mm^3^ samples and placed in phosphate-buffered saline (PBS) containing 0.5% (v/v) penicillin-streptomycin and 0.025% (v/v) amphotericin B. After the tissue was digested in Trypsin solution for 1.5 h at 37°C, the cell suspension was filtered through a 40-μm mesh (Thermo Fisher Scientific, Waltham, MA, USA) to remove the undigested material; then, the cells were resuspended in endometrial cell culture medium (DMEM-Ham's F-12 medium containing (Gibco, Grand Island, NY, USA), 10% fetal bovine serum (FBS, Gibco, Grand Island, NY, USA), 0.5% (v/v) penicillin-streptomycin, and 0.025% (v/v) amphotericin B). The cells were cultured in 25-cm^3^ flasks for 8 h for the selective attachment of the stromal cells. The cells were then maintained at 37°C in a humidified atmosphere with 5% CO_2_. Cells were immunostained as described previously (23). The bESC purification was confirmed as 99% via detection of vimentin-positive and cytokeratin-negative staining (Supplementary Figures 1, 2).

### Effect of FTA on bESC Viability

Cell viability was determined by colorimetric assay using a Cell Counting Kit-8 (CCK8, Solarbio, Beijing, China). Based on other related studies, we chose 12 h as the action time of FTA (17). In brief, bESCs (2 × 10^5^ cells/ml) were plated in triplicate in 96-well multiplies. Then, the cells were treated with FTA (0, 25, 50, 100, 200, 400, 600, 800, or 1,000 μg/ml) for 12 h followed by a 10 μl/well CCK8 treatment for 1 h. The absorbance was measured at 490 nm using a microplate reader. The FTA (HPLC ≥ 98%, NO. 79916-77-1) used in this study was provided by Chengdu Pufield Biotechnology Co., Ltd. (Pufield Biotechnology Co., Ltd, Chengdu, China) and was extracted from the dried fruit of *Forsythia suspensa* (*Forsythia suspensa* (Thunb.) Vahl), a plant belonging to the family Oleaceae.

### Cell Experiments

Our previous results showed that many important immune-related genes and signal pathways in the bESCs induced by LPS changed compared with the control group. In previous studies, the bESCs were stimulated using 0.5 μg/mL ultrapure LPS obtained from a pathogenic *E. coli* strain (serotype 055:B5, Sigma-Aldrich, Madison, USA, L2880) to mimic inflammatory conditions (24). In this study, we divided the experiment into LPS and LPS+FTA groups, each with three replicates (LPS1, LPS2, LPS3, FTA+LPS1, FTA+LPS2, FTA+LPS3). bESCs from passage 6 (P6) were shed in a 25-cm^2^ culture flask at a spatial arrangement of 2 × 10^5^ cells/ml. Upon reaching 80% confluence, bESCs were treated with 100 μg/mL FTA + 0.5 μg /mL LPS or 0.5 μg /mL LPS alone; cells were incubated for an additional 12 h.

### RNA Extraction, Small RNA Library Preparation, and Sequencing

Total RNA was extracted from samples in each experimental group using RNAiso plus reagent (TaKaRa, Shiga, Japan) according to the manufacturer's instructions. The quality and integrity of the isolated RNA were determined using Agilent 2100 Bioanalyser (Agilent Technologies). A NEBNext Multiplex Small RNA Library Prep Set for Illumina (New England Biolabs, Ipswich, USA) was used to construct small RNA libraries according to the manufacturer's instructions. Reverse transcription of the resulting samples was performed using Superscript II reverse transcriptase (TaKaRa, Shiga, Japan).

### Sequencing Data Processing

Unwanted sequences, including low-quality reads and those containing the 3′ adaptor, were removed to obtain clean data. The number of clean reads with sequence lengths of 18–36 nt was determined, and identical sequences in a single sample were deduplicated. Unique reads were mapped to the bovine genome (https://www.ncbi.nlm.nih.gov/genome/82?genome_assembly_id=371813) using miRDeep2 software (25). Then, small RNA (piRNA, rRNAs, tRNAs, snRNAs, snoRNA, and miRNA) sequences were annotated using piRBase, Rfam, and Repbase (http://www.girinst.org/education/index.html). Based on the genomic comparisons, novel miRNA prediction analysis was performed. According to the expression data of miRNA in each sample, the differential expression of miRNA was analyzed by DESeq (version 1.18.0) (26) using the following equation: log_2_ (fold change) > 1, and candidates with *P* < 0.05 were considered. The 3′ untranslated region (UTR) sequence of this species' mRNA was used as the target sequence to predict the target genes of miRNA sequences with differential expression using miRanda software.

### Gene Ontology (GO) and Kyoto Encyclopedia of Genes and Genomes (KEGG) Enrichment Analyses

According to molecular function (MF), biological process (BP), and cell component (CC), the predicted miRNA candidate target genes were analyzed by applying topGO (27) to reveal the functions of the differentially expressed miRNA target genes. KEGG pathway analysis was used to determine the functions of putative target genes regulated by miRNAs. *P* < 0.05 were considered as the significant demarcation point.

### Quantitative Real-Time PCR (qRT-PCR)

For verification of RNA-seq results, six miRNAs were randomly selected to perform quantitative real-time PCR (qRT-PCR). A cDNA synthesis kit (TaKaRa, Shiga, Japan) was used to reverse transcribe total RNA into cDNA following the manufacturer's instructions. QRT-PCR using the SYBR™ Green PCR Master Mix (TaKaRa, Shiga, Japan) was performed on a CFX96 real-time PCR detection system (Bio-Rad, Munich, Germany). The execution program of qRT-PCR was 40 cycles with denaturation at 95°C for 5 s and annealing at 60°C for 30 s. To normalize gene expression, the β*-actin* gene was used as an internal reference. The relative expression levels of the ten previously selected miRNA were analyzed using the 2^−ΔΔCt^ method (28). After normalizing the Ct value of the target gene and that of β*-actin*, the qRT-PCR data were analyzed. The qRT-PCR was performed in three biological replicates. Student's *t*-test was used to demonstrate the differences in mRNA expression between the samples. The statistical significance (*P* < 0.05) of the data was determined using Student's *t*-test with SPSS (PASW Statistics for Windows, Version 18.0; SPSS Inc., Chicago, IL, USA). The primers are listed in Table 1.

**Table 1:** List of real-time PCR primers used in the study.

**Gene**	**GenBank accession no**	**Forward primer 5^**′**^ - 3^**′**^**	**Reverse primer 5^**′**^ - 3^**′**^**
bta-miR-205	MIMAT0003545	GCCGTCCTTCATTCCACCGG	GTGCAGGGTCCGAGGT
bta-miR-677	MIMAT0012003	GCCGCTCACTGATGAGCAGCT	GTGCAGGGTCCGAGGT
bta-miR-708	MIMAT0009367	GCCGAAGGAGCTTACAATCTA	GTGCAGGGTCCGAGGT
bta-miR-497	MIMAT0004343	GCCGCAGCAGCACACTGTGG	GTGCAGGGTCCGAGGT
bta-miR-1468	MIMAT0013592	GCCGCTCCGTTTGCCTGTTT	GTGCAGGGTCCGAGGT
bta-miR-2388-5p	MIMAT0011940	GCCGAGCTCCCGTCTCCTCTG	GTGCAGGGTCCGAGGT
*β-actin*	NM_173979.3	AACTCCATCATGAAGTGTGACG	GATCCACATCTGCTGGAAGG

## Results

### Effect of FTA on bESC Viability

**Figure 2:**
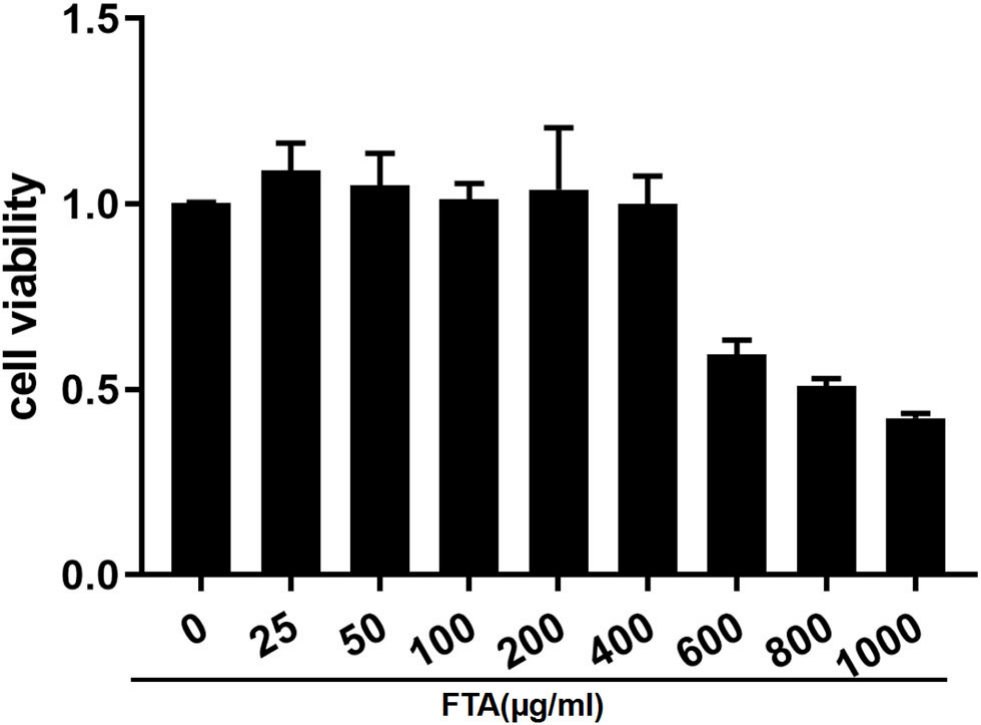
Effect of FTA on bESC viability. Cells were cultured with different concentrations of FTA (25, 50, 100, 200, 400, 600, 800, and 1,000 μg/ml) for 12 h. The cell viability was determined by CCK8 assay. The values presented are the means ± SEM.

To investigate the effects of FTA on bESC viability, a Cell Counting Kit-8 (CCK8, Solarbio, Beijing, China) assay was performed. As shown in Figure 2, FTA within 400 ug/ml had no significant effect on the survival rate of bESCs. However, the results demonstrate that when the concentration of FTA was 100 ug/ml, the effect on bESC activity was similar to that of the control group.

### Quality Control of the RNA

The RIN values of RNA samples, including LPS1, LPS2, LPS3, LPS+FTA1, LPS+FTA2, and LPS+FTA3, were 8.7, 8.5, 8.8, 8.5, 8.2, and 8.3, respectively (Supplementary Figure 3), indicating the RNA quality was fine.

### Sequence Analysis

To explore the function of differential miRNAs in the treatment of endometritis with FTA, we analyzed the expression pattern of miRNA in bESCs induced by LPS and treated with FTA by high-throughput sequencing. The average numbers of raw sequence reads (286, 621, 57.3, and 210, 592, 39.3) were obtained from six sRNA libraries (three LPS groups and three LPS+FTA groups, respectively). The average numbers of clean reads (251, 295, 91, and 129, 327, 30.3) were obtained from six sRNA libraries (three LPS groups and three LPS+FTA groups, respectively) after adapter trimming (Supplementary Table 1). Next, a principal component analysis was performed to determine the source of variance in the data; the result showed that three individual samples from each group were clustered closely together (Supplementary Figure 4). The unique reads were obtained by removing the same sequence in a single sample and counting the number of clean reads (i.e., the total read number) with lengths of 18–36n t. The read lengths were mainly distributed between 18 and 26 nt, and the peak was 23 nucleotides. According to the comparison results with the genome, we used MIREAP to analyze sequences without any annotated information to conduct novel miRNA prediction analysis. Novel miRNAs in each library with hairpin structures were obtained by prediction (Table 2 and Supplementary Table 2). Repeats, exons, introns, piRNA, and non-coding RNAs (miRNA, rRNA, tRNA, snoRNA, and snRNA) were annotated by mapping the unique reads to piRBase, Rfam, and Repbase databases (http://www.girinst.org/education/index.html) (Table 3). These data verified the high quality of the RNA-Seq analysis, which were further analyzed.

**Table 2:** List of novel miRNAs in six small RNA libraries of bESCs induced by lipopolysaccharide (LPS) and treated with forsythoside A (FTA).

**Sample**	**LPS1**	**LPS2**	**LPS3**	**LPS+FTA1**	**LPS+FTA2**	**LPS+FTA3**
Novel miRNA	93	92	104	64	58	67

**Table 3:** List of different sequence reads in the six small RNA libraries of bESCs induced by lipopolysaccharide (LPS) and treated with forsythoside A (FTA).

**Sample**	**LPS1**	**LPS2**	**LPS3**	**LPS+FTA1**	**LPS+FTA2**	**LPS+FTA3**
Known miRNA	20,488	20,678	19,875	16,043	15,427	18,600
piRNA	73,108	70,351	71,539	90,781	84,721	96,829
rRNA	1,584	1,628	1,537	3,212	2,923	3,188
tRNA	910	785	783	2,048	2,153	2,175
snRNA	97	66	99	57	64	151
snoRNA	1,246	911	1,216	901	1,025	1,916
repeat	331	270	293	458	559	662
exon	10,618	10,159	10,351	10,841	9,060	11,081
Intron	25,085	21,908	21,458	38,014	42,152	44,745
Novel miRNA	126	130	146	94	82	92
unknown	114,768	102,215	103,091	424,623	481,764	581,126

### Differentially Expressed miRNAs

**Figure 3:**
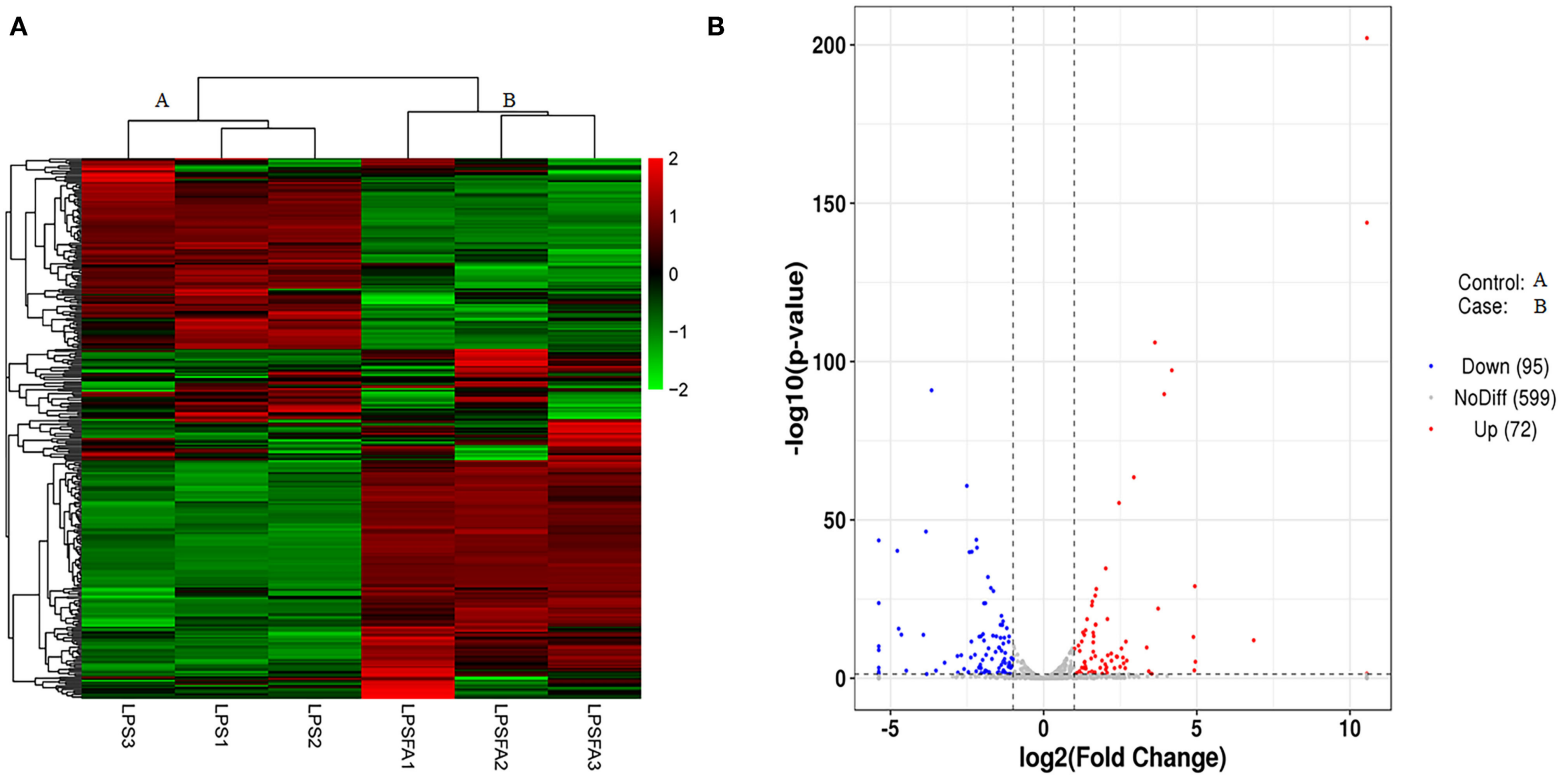
Comparison of miRNA expression patterns between lipopolysaccharide (LPS) and forsythoside A (FTA) + LPS groups. **(A)** Heat map of differentially expressed miRNAs. High and low expression levels of miRNA are shown in red and green, respectively. Each group included three biological repeats. **(B)** A volcano plot of differentially expressed miRNAs. Differences in miRNA up-regulation, down-regulation, and unchanged levels are represented by red, blue, and gray points, respectively.

The differential expression of miRNAs in each sample was analyzed using DESeq (26). A total of 167 miRNAs showed statistically significant differential expression between the LPS-only and LPS+FTA group [log_2_ (fold change) > 1 and *P* < 0.05 (Figure 3A and Supplementary Table 3)]. Of these, 72 miRNAs were up-regulated, and 95 were down-regulated. Among these miRNAs, the most up-regulated were bta-miR-205 (1,448.15-fold), bta-miR-1434-5p (115.36-fold), bta-miR-677 (18.13-fold), and bta-miR-2904 (30.90-fold), whereas the most down-regulated were bta-miR-497 (41.79-fold), bta-miR-195 (27.44-fold), bta-miR-142-5p (26.63-fold), and bta-miR-2284ab (15.22-fold). The target genes of the identified differentially expressed microRNA (DEGs) were predicted, and functional enrichment analysis of the target genes was conducted to determine the biological functions of the DEGs.

### GO and KEGG Pathway Analyses of the Target Genes

**Figure 4:**
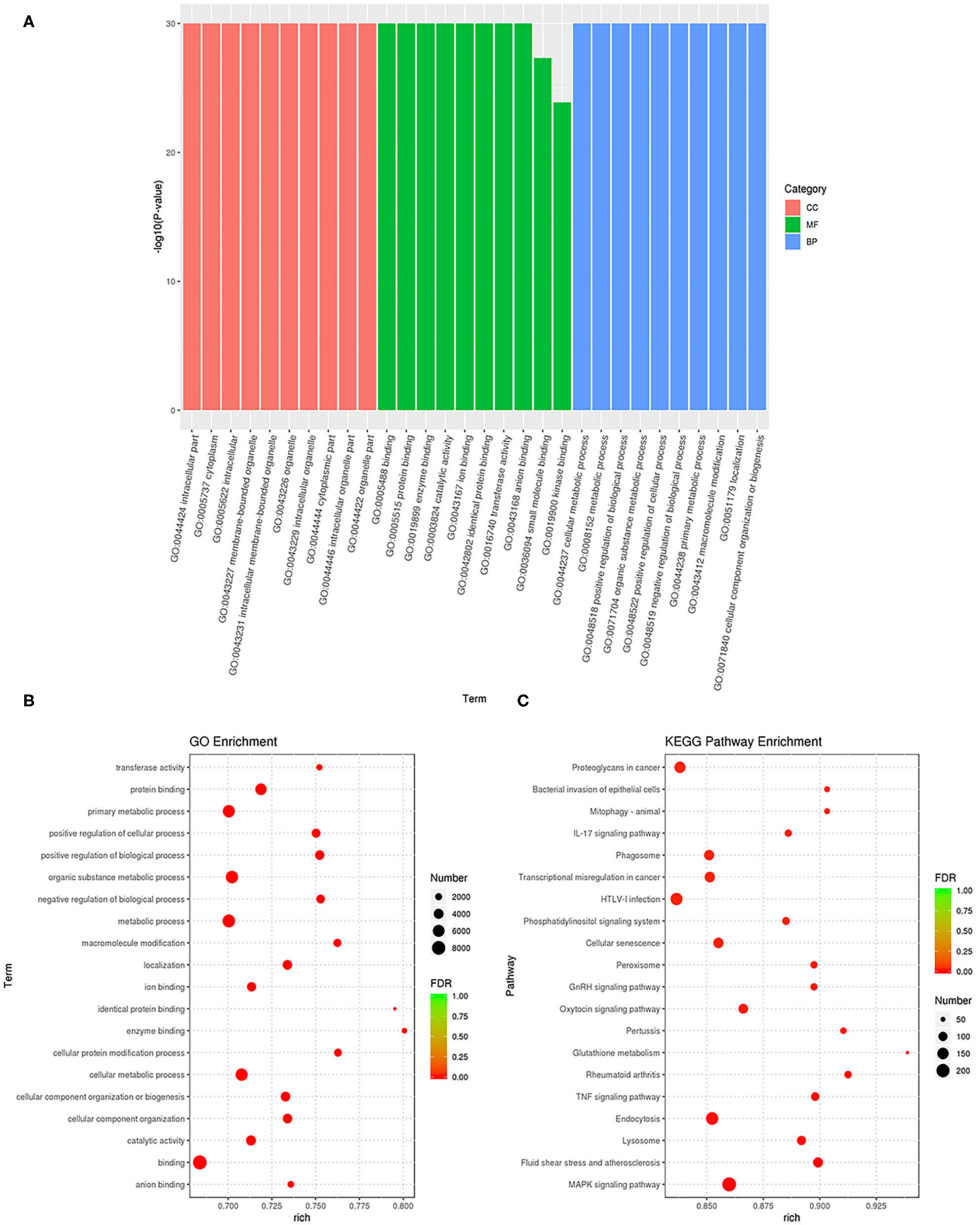
Functional annotation and enrichment analysis of differentially expressed miRNAs. **(A)** The results of Gene Ontology (GO) enrichment analysis of differentially expressed GO classified miRNA target genes according to molecular functional (MF), biological process (BP), and cellular component (CC) analysis, and the top 10 GO term items with the smallest *P*-value or the most significant enrichment were selected for display in each GO classification. The X-axis represents gene function, and the Y-axis represents gene percentage. **(B)** Scatter diagram of GO enrichment analysis of differentially expressed target genes of miRNA. **(C)** Scatter diagram of Kyoto Encyclopedia of Genes and Genomes (KEGG) pathway enrichment analysis of differentially expressed target genes of miRNA. The ratio of the number of differential miRNA target genes enriched in the pathway to the number of annotated differential miRNA target genes.

GO enrichment analysis was performed to annotate the target genes of these DEGs (Figure 4A and Supplementary Table 4). The results showed that in the BP analysis, the targets were mostly located in the cellular metabolic process, positive regulation of biological process, organic substance metabolic process, and positive regulation of cellular process. In the CC analysis, the targets were mostly attributed to the intracellular part, cytoplasm, and intracellular membrane-bounded organelles. The MF analysis showed that the targets mainly participated in the binding, protein binding, enzyme binding, catalytic activity, and ion binding. Also, we found that the gene percentages enriched in BP, CC, and MF were very high. The scatter plot shows the categories that had the most significant enrichment (Figure 4B). KEGG functional annotation was performed to predict the metabolic pathways that the target genes of these DEGs are involved in Supplementary Table 5. The top-20 KEGG pathways were the mitogen-activated protein kinase (MAPK) signaling pathway, fluid shear stress and atherosclerosis, lysosome, endocytosis, tumor necrosis factor (TNF) signaling pathway, rheumatoid arthritis, glutathione metabolism, pertussis, oxytocin signaling pathway, GnRH signaling pathway, peroxisome, cellular senescence, phosphatidylinositol signaling system, human T-cell leukemia virus type I (HTLV-I) infection, transcriptional misregulation in cancer, phagosome, IL-17 signaling pathway, mitophagy, bacterial invasion of epithelial cells, and proteoglycans in cancer (Figure 4C).

### Validation of the DEGs by RT-qPCR

**Figure 5:**
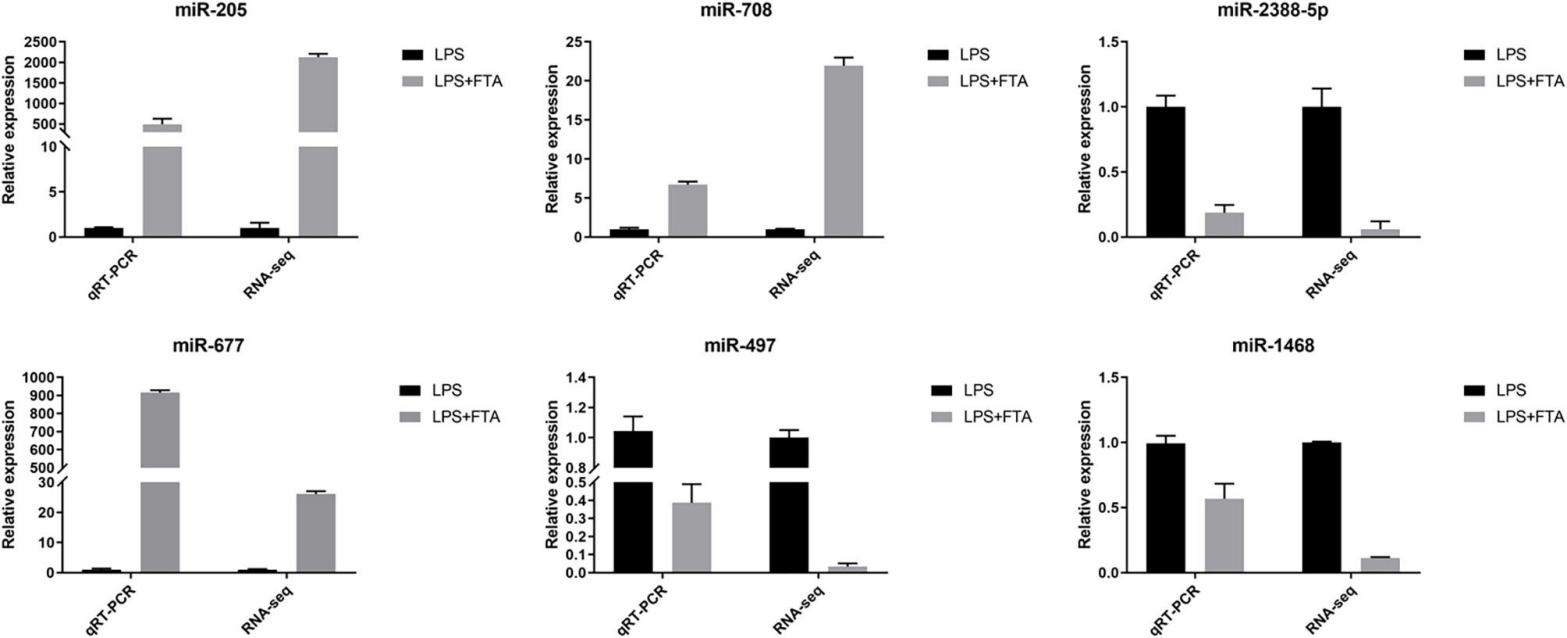
Validation of features of DEGs through qRT-PCR. The relative expression level of target miRNAs was calculated using the 2^−ΔΔCt^ method. Data represent the mean ± SEM (*n* = 3).The Log_2−_fold change was the average log_2−_fold change between samples.

Three up-regulated miRNAs bta-miR-205, bta-miR-677, and bta-miR-708 and three down-regulated miRNAsbta-miR-497, bta-miR-1468, and bta-miR-2388-5p were randomly selected from the library to perform RT-qPCR to verify the RNA-seq results. The RT-qPCR results showed that bta-miR-205, bta-miR-677, and bta-miR-708 were up-regulated in the LPS+FTA groups compared with the LPS group, and bta-miR-497, bta-miR-1468, and bta-miR-2388-5p were down-regulated in the LPS+FTA groups compared with the LPS groups. The RT-qPCR results were consistent with the RNA-seq results, thus confirming the RT-qPCR data (Figure 5).

## Discussion

Bovine postpartum endometritis caused by bacterial infection causes a substantial economic burden because it can delay the regeneration of endometrium, destroy the recovery of ovarian circulation (29), and lead to the failure of artificial insemination and infertility (30). The heavy use of antibiotics for the treatment of endometritis inevitably leads to the production of drug-resistant bacteria; thus, it is essential to develop a new, safe, and effective treatment strategy for endometritis. The ingredients of traditional medicinal plants may be useful to develop effective treatments against LPS-mediated toxicity. Previous studies have reported that FTA inhibits *Staphylococcus aureus*-stimulated inflammatory responses in bovine mammary epithelial cells by interfering with the activation of NF-κB and MAPK signaling pathways (31). FTA can exert an anti-inflammatory impact on LPS-induced BV2 microglial cells and primary microglial cells by inhibiting the activation of the NF-κB and Nrf2/HO-1 signaling pathways (18). The biological mechanism through which miRNAs modulate the inflammatory response induced by LPS has attracted broad attention. Overexpression of miR-223 can reduce the activation level of NOD-like receptor (NLRP3) to attenuate LPS-induced endometritis (32). NLRP3 inflammasome, a cytoplasmic protein complex that consists of NLRP3, caspase-1, and ASC, has been involved in the pathogenesis of multiple inflammatory diseases (33). The microRNA let-7c attenuates LPS-induced endometritis by inhibiting NF-κB signaling (34). MiR-19a also elicits an anti-in?ammatory e?ect and reduces the production of cytokines in LPS-induced endometritis by regulating the NF-κB pathway (35). The role of miRNA in the treatment of LPS-induced endometritis by FTA is unclear.

Altered miRNAs expression may play a role in inflammation by affecting target genes involved in different molecular networks and exerting various biological functions. The main purpose of this study was to reveal the mechanism underlying the effects of miRNAs in the treatment of endometritis with FTA. Therefore, we used LPS to stimulate bESCs to establish an *in vitro* anti-inflammatory model, and RNA sequencing was performed to monitor the effect of miRNAs on FTA in the treatment of endometritis.

Here, we identified 167 miRNAs that significantly differed between the LPS+FTA and LPS groups (log_2_ [fold change] > 1 and *P*-value < 0.05), including 72 up-regulated and 95 down-regulated miRNAs. These findings can help us better understand the possible molecular mechanism underlying miRNA action after LPS-induced endometritis treatment with FTA.

Endometrial stromal cells also express pattern recognition receptors similar to immune cells, including TLRs (36). Pathogen-associated molecules, such as LPS can activate TLRs, initiating a complex inflammatory response that results in the production and release of pro-inflammatory cytokines, type I interferons (IFNs), chemokines, and antibacterial proteins to clear the infection (37). In the present study, we found that bta-miRNA-146a up-regulates and targets TLR4. Epithelial and stromal cells respond to LPS by up-regulating the expression of TLR4 mRNA (38). Previous studies have found that LPS can up-regulate not only the expression of TLR4 but also the expression of TLR2. Here, we found that the up-regulated expression of bta-miR-29E may be related to TLR2. The high expression of TLR2 is related to the activation of the NF-κB-mitogen-activated protein kinase B (MAP3KB) complex and the up-regulation of pro-inflammatory cytokines and chemokines (39). In addition to membrane surface receptors, accumulating evidence suggests that LPS is recognized in cells in a TLR-independent manner, which triggers the activation of inflammatory proteases (40). Here, FTA treatment up-regulated the expression of bta-miR-677 (18.13-fold) and bta-miR-2904 (30.90-fold); these two miRNAs target DEXH Asp-Glu-X-His box polypeptide 58 (DHX58), which is involved in the intracellular recognition of pathogens or their ligands. These results suggest that FTA may regulate TLR4, TLR2, and DHX58 through miRNAs, reducing the inflammatory response induced by LPS.

Overexpression of inflammatory cytokines and chemokines can induce severe tissue damage and adversely affect pregnancy (1). Several studies have found that the mRNAs of interleukin (IL) 1A, IL1B, IL6, IL8, chemokine CXL ligand (CXCL) 3, and CXCL5 are up-regulated in dairy cows with endometritis (39, 41, 42). To some extent, miRNAs can regulate the expression and release of pro-inflammatory and anti-inflammatory factors. Here, we found that bta-miR-205 was the most up-regulated miRNA induced by FTA, with an ~1,448.15-fold increase in expression. Moreover, we showed that bta-miR-205 targeted several inflammatory cytokines, including IL1A, IL27, IL2RA, and IL21. In particular, the expression of pro-inflammatory cytokine IL1A mRNA was significantly increased in endometritis (43). In addition, the sequencing results show that IL1A is another target gene of the significantly up-regulated bta-miR-2904. Furthermore, we showed that IL1B was a target gene of bta-miR-31. Bta-miR-31 expression was 7.46-fold higher in the FTA treatment group. IL6ST is a signal transducer of IL-6 and a target gene of bta-miR-302a, bta-miR-302b, and bta-miR302-d (44). Bta-miR-1434-5p was the second most up-regulated miRNA in the FTA group.

**Figure 6:**
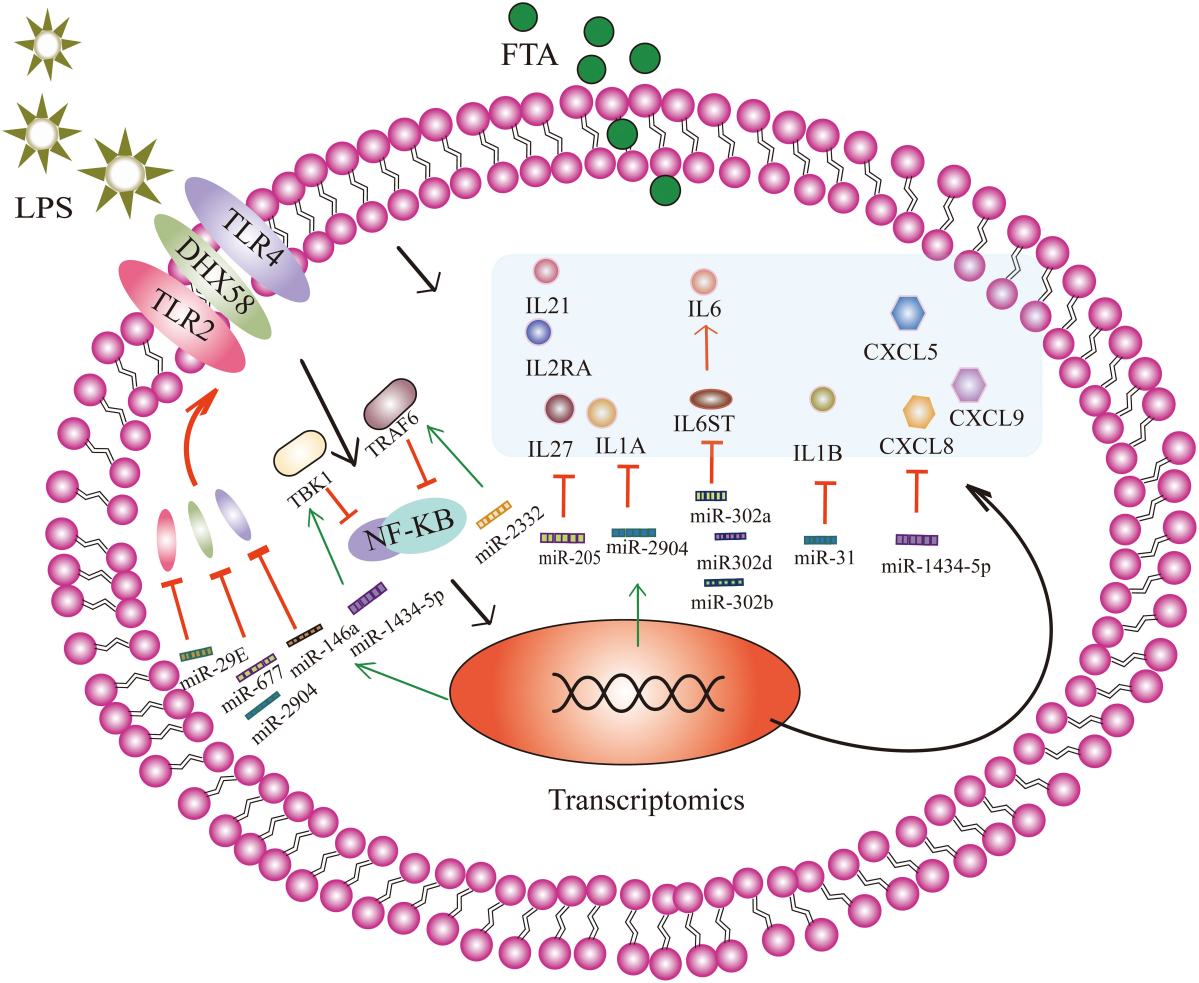
Forsythoside A (FTA) attenuates lipopolysaccharide (LPS)-induced endometritis by causing significant changes in the number of miRNAs in bESCs to regulate the target genes involved in different pathway. Through the activation of the NF-κB signaling pathway, a complex inflammatory response occurs, and pro-inflammatory cytokines and chemokines are produced and released. FTA can increase the expression of miRNA-146a, miR-29E, miR-677, and miR-2904, and negatively regulate the target genes, TLR4, TRL2, and DHX58, to reduce the production of TLR4, TRL2, and DHX58 receptor proteins. MiR-1434-5p and miR-2332 can attenuate the activation of the NF-κB signaling pathway by targeting TBK1 and TRAF6, respectively. MiR-205, miR-2904, miR-31, miR-302a, miR-302b, miR302-d, and miR-1434-5p target multiple cytokines and chemokines to reduce the production of inflammatory factors and reduce the inflammatory response. For example, bta-miR-205 targeted several inflammatory cytokines, including IL1A, IL27, IL2RA, and IL21. Bta-miR-2904 targeted IL1A. IL6ST is a signal transducer of IL-6 and a target gene of bta-miR-302a, bta-miR-302b, and bta-miR302-d. Bta-miR-1434-5p targeted chemokines CXCL5, CXCL8, and CXCL9. IL1B is a target gene of bta-miR-31. The black arrow indicates the mechanism underlying the inflammatory response of bESCs stimulated by LPS, and the green arrow indicates that miRNA expression partly increases under the action of FTA. The red T-shaped line indicates that miRNA targets inflammatory cytokines and reduces the expression of inflammatory cytokines, and red arrows indicate that inflammatory factors or proteins can promote the expression of downstream inflammatory factors or proteins.

Bta-miR-1434-5p was up-regulated 115.36-fold and targeted chemokines CXCL5, CXCL8, and CXCL9. We speculated that FTA treatment might up-regulate bta-miR-1434-5p to control the expression of CXCL5, CXCL8, and CXCL9. In addition, we found that RSAD2 was one of the predictive target genes of bta-miR-142-5p. RSAD2 (viperin) is a member of type I interferon (IFN)-stimulated genes (ISGs), and is considered to have antiviral and antibacterial activities (45). Figure 6 shows the miRNAs that are highly expressed and their target genes involved in anti-inflammatory regulation. The expression of pro-inflammatory mediators affects embryonic development through the action of oocytes and endometrial tissue, resulting in the termination of pregnancy in cattle (1). These results indicated that the anti-inflammatory effect of FTA in endometritis might be related to the regulation of inflammatory cytokine and chemokine expression by miRNAs. Besides, the mechanism through which these miRNAs regulate changes in inflammatory cytokines and chemokines require further studies using luciferase reporter assays and RNA binding protein immunoprecipitation. By analyzing the relationship between these differential changes in miRNAs, chemokines, and inflammatory factors, we can develop more methods for treating endometritis from the perspective of miRNAs, and reveal the complex mechanisms underlying the action of miRNAs and target genes in the process of anti-inflammation.

A previous study showed that TBK1 is highly expressed in an LPS-induced hepatitis model (46). As a member of the IκB kinase (IKK) family, TBK1 regulates inflammatory cytokines expression and plays an important role in regulating immune responses induced by bacterial and viral infections (47, 48). Also, Yin et al. (35). Suggested that miR-19a can attenuate the activation of the NF-κB signaling pathway by targeting TBK1, thus reducing the production of inflammatory factors and inhibiting the inflammatory response of endometritis induced by LPS. In this study, we found no significant change in the expression of miR-19a; however, TBK1 was one of the target genes of bta-mir-1434-5p, which was identified and showed 115.36-fold high expression. We speculate that FTA may regulate LPS-induced inflammation by regulating bta-miR-1434-5p targeting of TBK1. In addition, TRAF6 is also involved in the activation of the NF-κB signaling pathway and promotes inflammatory responses (49, 50). Zhao et al. revealed that miR-643 inhibits the LPS-induced inflammatory response by targeting TRAF6 and down-regulating TRAF6 in human endometrial epithelial cells (51). In our study, TRAF6 was one of the target genes up-regulated by 6.32 folds by bta-miR-2332. We suggest that FTA alleviates LPS-induced endometritis and may be related to the targeted regulation of TRAF6 by bta-miR-2332.

The target genes of 167 differentially expressed miRNAs were selected for GO and KEGG analyses. The MAPK, TNF, and IL-17 signaling pathways were significantly enriched in KEGG analysis. In addition, KEGG analysis showed that the MAPK signaling pathway was the most significantly enriched category. The MAPK pathways include extracellular signal-regulated kinase 1/2 (ERK1/2), p38, and c-Jun NH2-terminal kinase (JNK). MAPK pathways are reported to be involved in regulating the LPS-induced expression of TNF-α (52). Previous studies demonstrated that ferulic acid and cortisol could inhibit LPS-induced inflammation by suppressing the NF-κB and MAPK pathways in bovine endometrial epithelial cells (53, 54). Therefore, we speculate that FTA may also play an anti-inflammatory effect through the MAPK signaling pathway.

## Conclusions

In summary, to the best of our knowledge, this is the first study to use high-throughput RNA sequencing (RNA-seq) to comprehensively analyse the miRNA expression pattern in LPS-induced bESCs treated with FTA. Our study provides a detailed understanding of the molecular mechanism underlying the attenuation of LPS-induced endometritis by FTA. We suggest that FTA may attenuate LPS-induced endometritis by regulating inflammatory factors and chemokines through miRNAs. Multiple pathways related to immune function were found to be significantly enriched. These differentially expressed miRNAs may provide a new therapeutic direction for attenuating LPS-induced endometritis. Limitations of this study include that RNA sequencing technology only allows us for the identification of crucial miRNAs but not their specific roles. Additionally, further studies of the FTA anti-inflammatory mechanism are needed.

## Data Availability Statement

The data presented in the study are deposited in the https://www.ncbi.nlm.nih.gov/ repository, accession number PRJNA612133.

## Ethics Statement

Ethical approval was obtained from the Institutional Animal Use Committee of Henan Agricultural University (approval number. 2005-0026) and Beijing Association for Science and Technology (approval SYXK [Beijing] 2007-0023).

## Author Contributions

XW, CT, and HL designed the study and revised the manuscript. HL, CY, LD, WH, ZP, XD, and DY performed the study. HL, CY, CT, and XW analyzed the data. HL, CY, and DL wrote the manuscript. All authors read and approved the final manuscript.

## Conflict of Interest

The authors declare that the research was conducted in the absence of any commercial or financial relationships that could be construed as a potential conflict of interest.

## Supplementary Material

Supplementary Figure 1 Morphological characteristics of P3 bESCs. bESCs were fibroblast-like and enlarged at the time of confluence after which they overgrew in multiple layers (Scale bars =100 μm, 100 ×).

Supplementary Figure 2 Immunofluorescence staining shows the positive expression of vimentin expression (green) and negative expression of cytokeratin-18 (red) in bESCs (Scale bars = 50 μm, 100×). **(A)** Nuclear staining with DAPI (blue) of bESCs. **(B)** Immunostaining images of vimentin (green) of bESCs. **(C)** Immunofluorescence of bESCs. **(D)** Nuclear staining with DAPI (blue) of bESCs. **(E)** Immunostaining images of keratin-18 (red) of bESCs. **(F)** Immunofluorescence of bESCs.

Supplementary Figure 3 The RIN values of RNA including LPS 1, LPS 2, LPS 3, LPS+FTA 1, LPS+FTA 2, LPS+FTA 3. **(A)** The RIN values of RNA in LPS1 group. **(B)** The RIN values of RNA in LPS2 group. **(C)** The RIN values of RNA in LPS3 group. **(D)** The RIN values of RNA in LPS+FTA1 group. **(E)** The RIN values of RNA in LPS+FTA2 group. **(F)** The RIN values of RNA in LPS+FTA3 group.

Supplementary Figure 4 Principal Component Analysis **(**PCA) of transcriptomes of all samples.

Supplementary Table 1 The number of unique reads for each sample.

Supplementary Table 2 List of novel miRNAs in six small RNA libraries.

Supplementary Table 3 List of significantly differentially expressed miRNAs.

Supplementary Table 4 Gene Ontology (GO) enrichment analysis of the differentially expressed miRNA target genes.

Supplementary Table 5 Kyoto Encyclopedia of Genes and Genomes (KEGG) enrichment analysis of the differentially expressed miRNA target genes.

## Abbreviations

FTA: Forsythoside A
LPS: Lipopolysaccharide
BBESs: bovine endometrial stromal cells
BBECs: Bovine endometrial epithelial cells
miRNAs: MicroRNAs.

## References

[1] Sheldon IM Lewis GS LeBlanc S Gilbert RO. Defining postpartum uterine disease in cattle. Theriogenology. (2006) 65:1516–30. 10.1016/j.theriogenology.2005.08.021 16226305

[2] Ordell A Unnerstad HE Nyman A Gustafsson H Bage R. A longitudinal cohort study of acute puerperal metritis cases in Swedish dairy cows. Acta Vet Scand. (2016) 58:79. 10.1186/s13028-016-0257-9 27832812 PMC5105271

[3] Sheldon IM Cronin J Goetze L Donofrio G Schuberth HJ. Defining postpartum uterine disease and the mechanisms of infection and immunity in the female reproductive tract in cattle. Biol Reprod. (2009) 81:1025–32. 10.1095/biolreprod.109.077370 19439727 PMC2784443

[4] Sheldon IM Molinari PCC Ormsby TJR Bromfield JJ. Preventing postpartum uterine disease in dairy cattle depends on avoiding, tolerating and resisting pathogenic bacteria. Theriogenology. (2020) 150:158–65. 10.1016/j.theriogenology 31973964 PMC7234917

[5] Cronin JG Turner ML Goetze L Bryant CE Sheldon IM. Toll-like receptor 4 and MYD88-dependent signaling mechanisms of the innate immune system are essential for the response to lipopolysaccharide by epithelial and stromal cells of the bovine endometrium. Biol Reprod. (2012) 86:51. 10.1095/biolreprod.111.092718 22053092 PMC4396703

[6] Turner ML Cronin JG Healey GD Sheldon IM. Epithelial and stromal cells of bovine endometrium have roles in innate immunity and initiate inflammatory responses to bacterial lipopeptides *in vitro* via Toll-like receptors TLR2, TLR1, and TLR6. Endocrinology. (2014) 155:1453–65. 10.1210/en.2013-1822 24437488 PMC3959608

[7] Chapwanya A Meade KG Doherty ML Callanan JJ Mee JF O'Farrelly C. Histopathological and molecular evaluation of Holstein-Friesian cows postpartum: toward an improved understanding of uterine innate immunity. Theriogenology. (2009) 71:1396–407. 10.1016/j.theriogenology.2009.01.006 19233457

[8] Wagner WC Hansel W. Reproductive physiology of the postpartum cow. I. Clinical and histological findings. J Reprod Fertil. (1969) 18:493–500. 10.1530/jrf.0.0180493 5788219

[9] Ireland JJ Murphee RL Coulson PB. Accuracy of predicting stages of bovine estrous cycle by gross appearance of the corpus luteum. J Dairy Sci. (1980) 63:155–60. 10.3168/jds.S0022-0302(80)82901-8 7372895

[10] Bartel DP. MicroRNAs: genomics, biogenesis, mechanism, and function. Cell. (2004) 116:281–97. 10.1016/s0092-8674(04)00045-5 14744438

[11] Ambros V. The functions of animal microRNAs. Nature. (2004) 431:350–5. 10.1038/nature02871 15372042

[12] Xie B Lu C Chen C Zhou J Deng Z. MiR-135a alleviates silica-induced pulmonary fibrosis by targeting NF-kappaB/inflammatory signaling pathway. Media Inflamm. (2020) 2020:1231243. 10.1155/2020/1231243 32617074 PMC7317310

[13] Yan Y Lu K Ye T Zhang Z. MicroRNA-223 attenuates LPS-induced inflammation in an acute lung injury model via the NLRP3 inflammasome and TLR4/NF-κB signaling pathway via RHOB. Int J Mol Med. (2019) 43:1467–77. 10.3892/ijmm.2019.4075 30747229 PMC6365085

[14] Li S Xie R Jiang C Liu M. Schizandrin a alleviates LPS-induced injury in human keratinocyte cell hacat through a microRNA-127-Dependent regulation. Cell Physiol Biochem. (2018) 49:2229–39. 10.1159/000493826 30257250

[15] Xu L Huang R. Textual research on “Lianqiao” whose other name be “Lanhua” (cymbidium) in Shen Nong's Herbal. Lishizhen Med Res. (2000) 11:358–9.

[16] Gong LYL Hu N Li Y. Advances on the anti-inflammatory material basis of lianqiao and its action mechanism. Pharma Chi Med. (2019) 10:43–9.

[17] Qian J Ma X Xun Y Pan L. Protective effect of forsythiaside A on OVA-induced asthma in mice. Eur J Pharmacol. (2017) 812:250–5. 10.1016/j.ejphar.2017.07.033 28733217

[18] Wang Y Zhao H Lin C Ren J Zhang S. Forsythiaside A exhibits anti-inflammatory effects in LPS-stimulated BV2 microglia cells through activation of Nrf2/HO-1 signaling pathway. Neurochem Res. (2016) 41:659–65. 10.1007/s11064-015-1731-x 26498935

[19] Pan CW Zhou GY Chen WL Zhuge L Jin LX Zheng Y. Protective effect of forsythiaside A on lipopolysaccharide/d-galactosamine-induced liver injury. Int Immunopharmacol. (2015) 26:80–5. 10.1016/j.intimp.2015.03.009 25797347

[20] Zeng XY Yuan W Zhou L Wang SX Xie Y Fu YJ. Forsythoside A exerts an anti-endotoxin effect by blocking the LPS/TLR4 signaling pathway and inhibiting tregs *in vitro*. Int J Mol Med. (2017) 40:243–50. 10.3892/ijmm.2017.2990 28534936

[21] Fortier MA Guilbault LA Grasso F. Specific properties of epithelial and stromal cells from the endometrium of cows. J Reprod Fertil. (1988) 83:239–48. 10.1530/jrf.0.0830239 3165129

[22] Jacca S Franceschi V Colagiorgi A Sheldon M Donofrio G. Bovine endometrial stromal cells support tumor necrosis factor alpha-induced bovine herpesvirus type 4 enhanced replication. Biol Reprod. (2013) 88:135. 10.1095/biolreprod.112.106740 23515672

[23] Jacca S Franceschi V Agosti M Cavirani S Mistretta F Donofrio G. Interferon gamma-mediated BoHV-4 replication restriction in bovine endometrial stromal cells is host IDO1 gene expression independent and BoHV-4 IE2 gene expression dependent. Biol Reprod. (2014) 91:112. 10.1095/biolreprod.114.123000 25273529

[24] Ding X Lv H Deng L Hu W Peng Z Yan C. Analysis of transcriptomic changes in bovine endometrial stromal cells treated with lipopolysaccharide. Front Vet Sci. (2020) 7:575865. 10.3389/fvets.2020.575865 33324700 PMC7725876

[25] Mackowiak SD. Identification of novel and known miRNAs in deep-sequencing data with miRDeep2. Curr Protoc Bioinformatics. (2011) 12:10. 10.1002/0471250953.bi1210s36 22161567

[26] Anders S Huber W. Differential expression analysis for sequence count data. Genome Biol. (2010) 11:R106. 10.1186/gb-2010-11-10-r106 20979621 PMC3218662

[27] Alexa A Rahnenfuhrer J Lengauer T. Improved scoring of functional groups from gene expression data by decorrelating GO graph structure. Bioinformatics. (2006) 22:1600–07. 10.1093/bioinformatics/btl140 16606683

[28] Mehmood K Zhang H Iqbal MK Rehman MU Shahzad M Li K. *In vitro* effect of apigenin and danshen in tibial dyschondroplasia through inhibition of heat-shock protein 90 and vascular endothelial growth factor expressions in avian growth plate cells. Avian Dis. (2017) 61:372–7. 10.1637/11641-032817-RegR 28957003

[29] Sicsic R Goshen T Dutta R Kedem-Vaanunu N Kaplan-Shabtai V Pasternak Z. Microbial communities and inflammatory response in the endometrium differ between normal and metritic dairy cows at 5-10 days post-partum. Vet Res. (2018) 49:77. 10.1186/s13567-018-0570-6 30068391 PMC6071394

[30] Machado Pfeifer LF de Souza Andrade J Moreira EM Reis da Silva R Araujo Neves PM Moreira da Silva G. Uterine inflammation and fertility of beef cows subjected to timed AI at different days postpartum. Anim Reprod Sci. (2018) 197:268–77. 10.1016/j.anireprosci.2018.08.039 30195943

[31] Zhang J Zhang Y Huang H Zhang H Lu W Fu G. Forsythoside A inhibited *S. aureus* stimulated inflammatory response in primary bovine mammary epithelial cells. Microb Pathog. (2018) 116:158–63. 10.1016/j.micpath.2018.01.002 29330061

[32] Zhao G Jiang K Yang Y Zhang T Wu H Shaukat A. The potential therapeutic role of miR-223 in bovine endometritis by targeting the NLRP3 inflammasome. Front Immunol. (2018) 9:1916. 10.3389/fimmu.2018.01916 30186287 PMC6113393

[33] Yan Y Jiang W Liu L Wang X Ding C Tian Z. Dopamine controls systemic inflammation through inhibition of NLRP3 inflammasome. Cell. (2015) 160:62–73. 10.1016/j.cell.2014.11.047 25594175

[34] Zhao G Zhang T Wu H Jiang K Qiu C Deng G. MicroRNA let-7c improves LPS-induced outcomes of endometritis by suppressing NF-κB signaling. Inflammation. (2019) 42:650–7. 10.1007/s10753-018-0922-4 30406463

[35] Yin N Yang Y Wang X Yang C Ma X Shaukat A. MiR-19a mediates the negative regulation of the NF-κB pathway in lipopolysaccharide-induced endometritis by targeting TBK1. Inflamm Res. (2019) 68:231–40. 10.1007/s00011-019-01213-3 30673803

[36] Davies D Meade KG Herath S Eckersall PD Gonzalez D White JO. Toll-like receptor and antimicrobial peptide expression in the bovine endometrium. Reprod Biol Endocrinol. (2008) 6:53. 10.1186/1477-7827-6-53 19017375 PMC2627908

[37] Takeuchi O Akira S. Pattern recognition receptors and inflammation. Cell. (2010) 140:805–20. 10.1016/j.cell.2010.01.022 20303872

[38] Herath S Fischer DP Werling D Williams EJ Lilly ST Dobson H. Expression and function of Toll-like receptor 4 in the endometrial cells of the uterus. Endocrinology. (2006) 147:562–70. 10.1210/en.2005-1113 16223858 PMC2738982

[39] Oguejiofor CF Cheng Z Abudureyimu A Fouladi-Nashta AA Wathes DC. Global transcriptomic profiling of bovine endometrial immune response *in vitro*. I. Effect of lipopolysaccharide on innate immunity. Biol Reprod. (2015) 93:100. 10.1095/biolreprod.115.128868 26353891

[40] Kayagaki N Wong MT Stowe IB Ramani SR Gonzalez LC Akashi-Takamura S. Noncanonical inflammasome activation by intracellular LPS independent of TLR4. Science. (2013) 341:1246–9. 10.1126/science.1240248 23887873

[41] Peter S Michel G Hahn A Ibrahim M Lubke-Becker A Jung M. Puerperal influence of bovine uterine health status on the mRNA expression of pro-inflammatory factors. J Physiol Pharmacol. (2015) 66:449–62. 26084227

[42] Gabler C Fischer C Drillich M Einspanier R Heuwieser W. Time-dependent mRNA expression of selected pro-inflammatory factors in the endometrium of primiparous cows postpartum. Reprod Biol Endocrinol. (2010) 8:152. 10.1186/1477-7827-8-152 21176181 PMC3016299

[43] Fischer C Drillich M Odau S Heuwieser W Einspanier R Gabler C. Selected pro-inflammatory factor transcripts in bovine endometrial epithelial cells are regulated during the oestrous cycle and elevated in case of subclinical or clinical endometritis. Reprod Fertil Dev. (2010) 22:818–29. 10.1071/rd09120 20450834

[44] Hibi M Murakami M Saito M Hirano T Taga T Kishimoto T. Molecular cloning and expression of an IL-6 signal transducer, gp130. Cell. (1990) 63:1149–57. 10.1016/0092-8674(90)90411-7 2261637

[45] Severa M Coccia EM Fitzgerald KA. Toll-like receptor-dependent and -independent viperin gene expression and counter-regulation by PRDI-binding factor-1/BLIMP1. J Biol Chem. (2006) 281:26188–95. 10.1074/jbc.M604516200 16849320

[46] Marchlik E Thakker P Carlson T Jiang Z Ryan M Marusic S. Mice lacking Tbk1 activity exhibit immune cell infiltrates in multiple tissues and increased susceptibility to LPS-induced lethality. J Leukoc Biol. (2010) 88:1171–80. 10.1189/jlb.0210071 20651301

[47] Yu T Yi YS Yang Y Oh J Jeong D Cho JY. The pivotal role of TBK1 in inflammatory responses mediated by macrophages. Mediators Inflamm. (2012) 2012:979105 10.1155/2012/979105 23304064 PMC3523167

[48] Xie XH Zang N Li SM Wang LJ Deng Y He Y. Resveratrol inhibits respiratory syncytial virus-induced IL-6 production, decreases viral replication, and downregulates TRIF expression in airway epithelial cells. Inflammation. (2012) 35:1392–401. 10.1007/s10753-012-9452-7 22391746

[49] Cui Y Wang Y Zhao D Feng X Zhang L Liu C. Loganin prevents BV-2 microglia cells from Abeta1-42 -induced inflammation via regulating TLR4/TRAF6/NF-kappaB axis. Cell Biol Int. (2018) 42:1632–42. 10.1002/cbin.11060 30288860

[50] Liu K Chen K Zhang Q Zhang L Yan Y Guo C. TRAF6 neddylation drives inflammatory arthritis by increasing NF-kappaB activation. Lab Invest. (2019) 99:528–38. 10.1038/s41374-018-0175-8 30626891 PMC6484715

[51] Zhao R Wang J Zhang X Chen Y. MiR-643 inhibits lipopolysaccharide-induced endometritis progression by targeting TRAF6. Cell Biol Int. (2020) 44:1059–67. 10.1002/cbin.11306 31930635

[52] Yoon WJ Lee NH Hyun CG. Limonene suppresses lipopolysaccharide-induced production of nitric oxide, prostaglandin E2, and pro-inflammatory cytokines in RAW 264.7 macrophages. J Oleo Sci. (2010) 59:415–21. 10.5650/jos.59.415 20625233

[53] Dong J Qu Y Li J Cui L Wang Y Lin J. Cortisol inhibits NF-κB and MAPK pathways in LPS activated bovine endometrial epithelial cells. Int Immunopharmacol. (2018) 56:71–7. 10.1016/j.intimp.2018.01.021 29367089

[54] Yin P Zhang Z Li J Shi Y Jin N Zou W. Ferulic acid inhibits bovine endometrial epithelial cells against LPS-induced inflammation via suppressing NK-κB and MAPK pathway. Res Vet Sci. (2019) 126:164–9. 10.1016/j.rvsc.2019.08.018 31499425

