# Supplementary figures and images for: Role of MicroRNAs in Protective Effects of Forsythoside A Against Lipopolysaccharide-Induced Inflammation in Bovine Endometrial Stromal Cells
Source: Front Vet Sci. 2021 Feb 24;8:642913. doi: 10.3389/fvets.2021.642913 (PMC7943879; doi:10.3389/fvets.2021.642913)

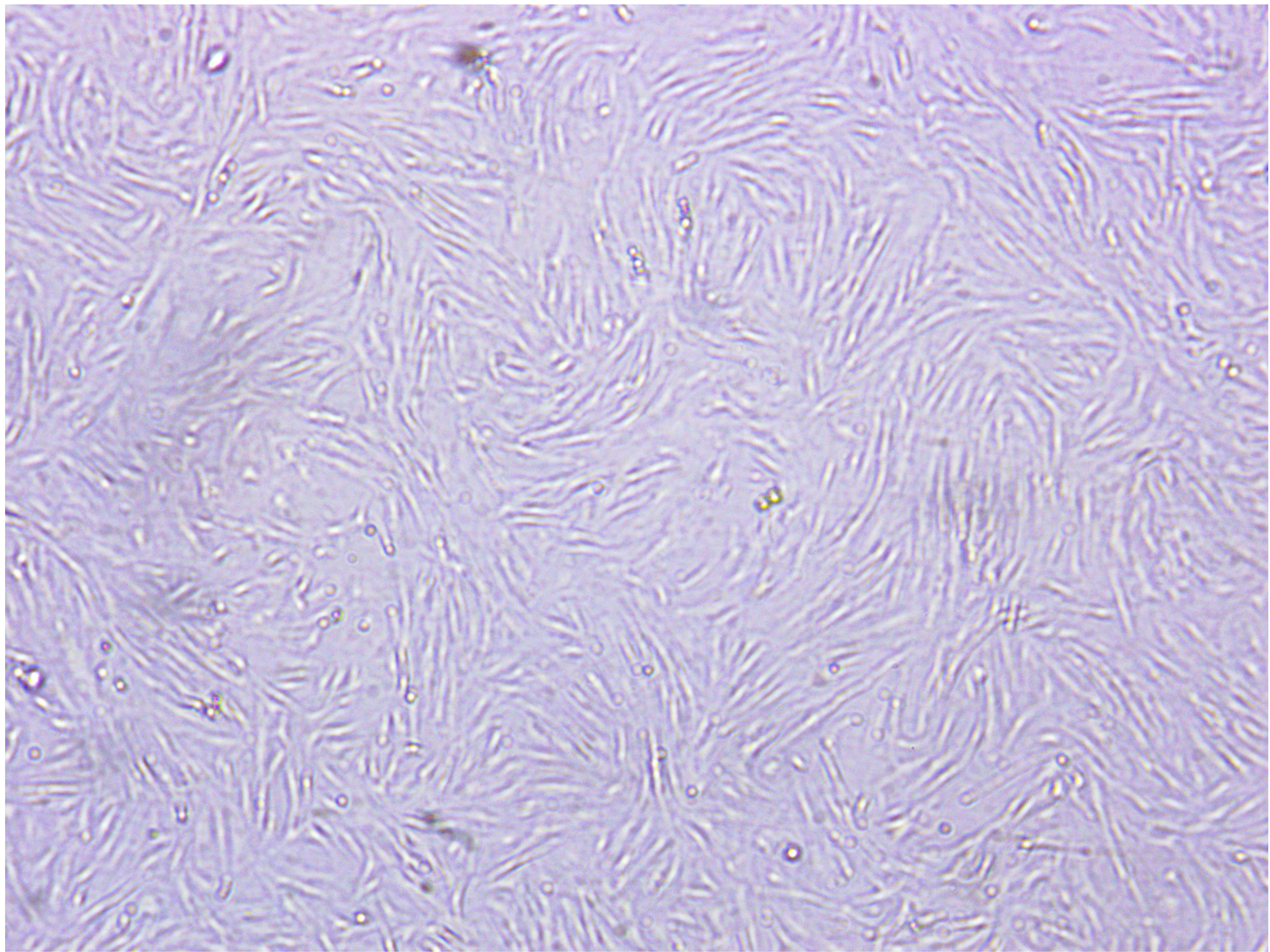

Supplement: Supplementary Figure 1 — Morphological characteristics of P3 bESCs. bESCs were fibroblast-like and enlarged at the time of confluence after which they overgrew in multiple layers (Scale bars =100 μm, 100 ×). [file Data_Sheet_1.PDF]

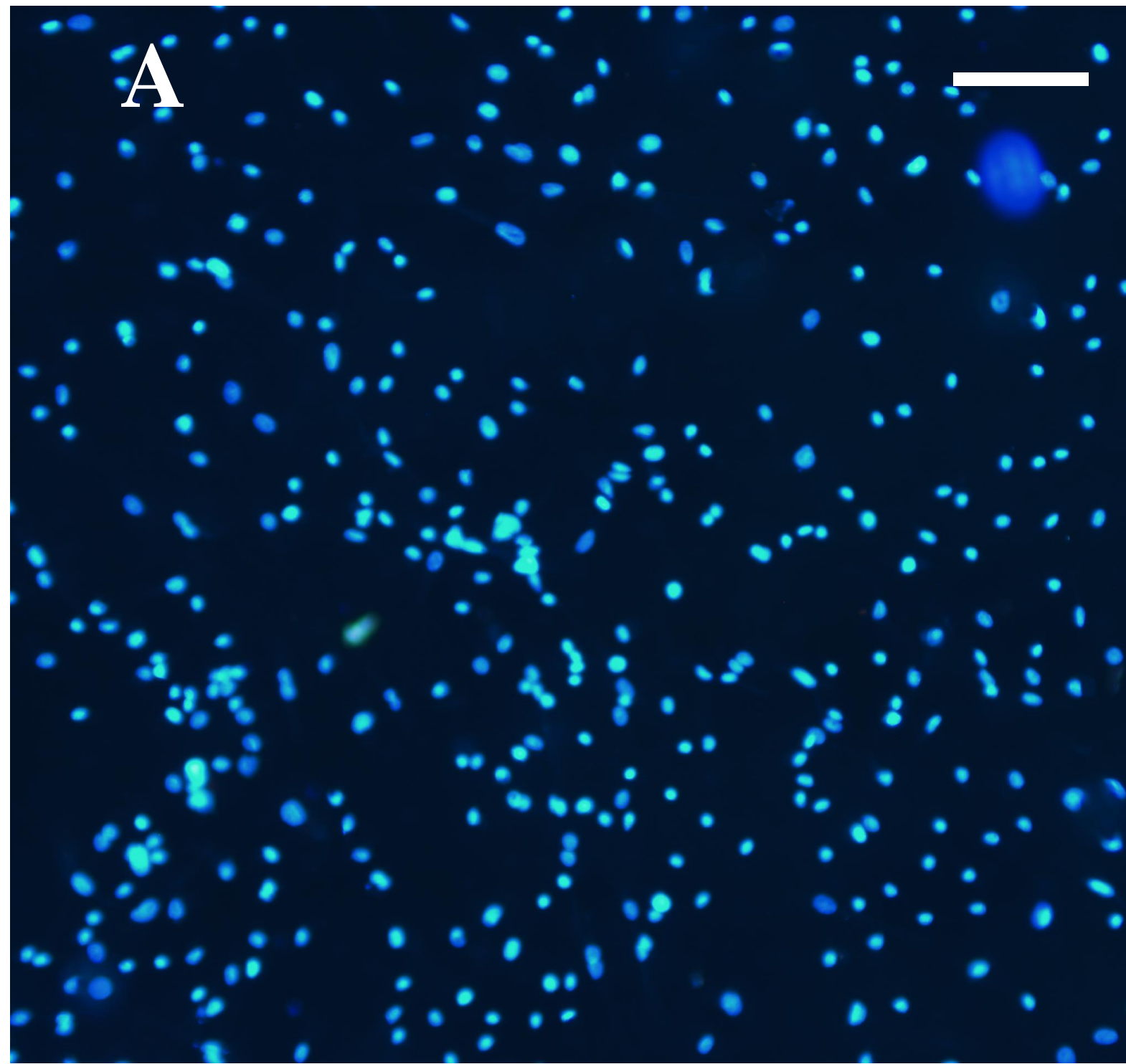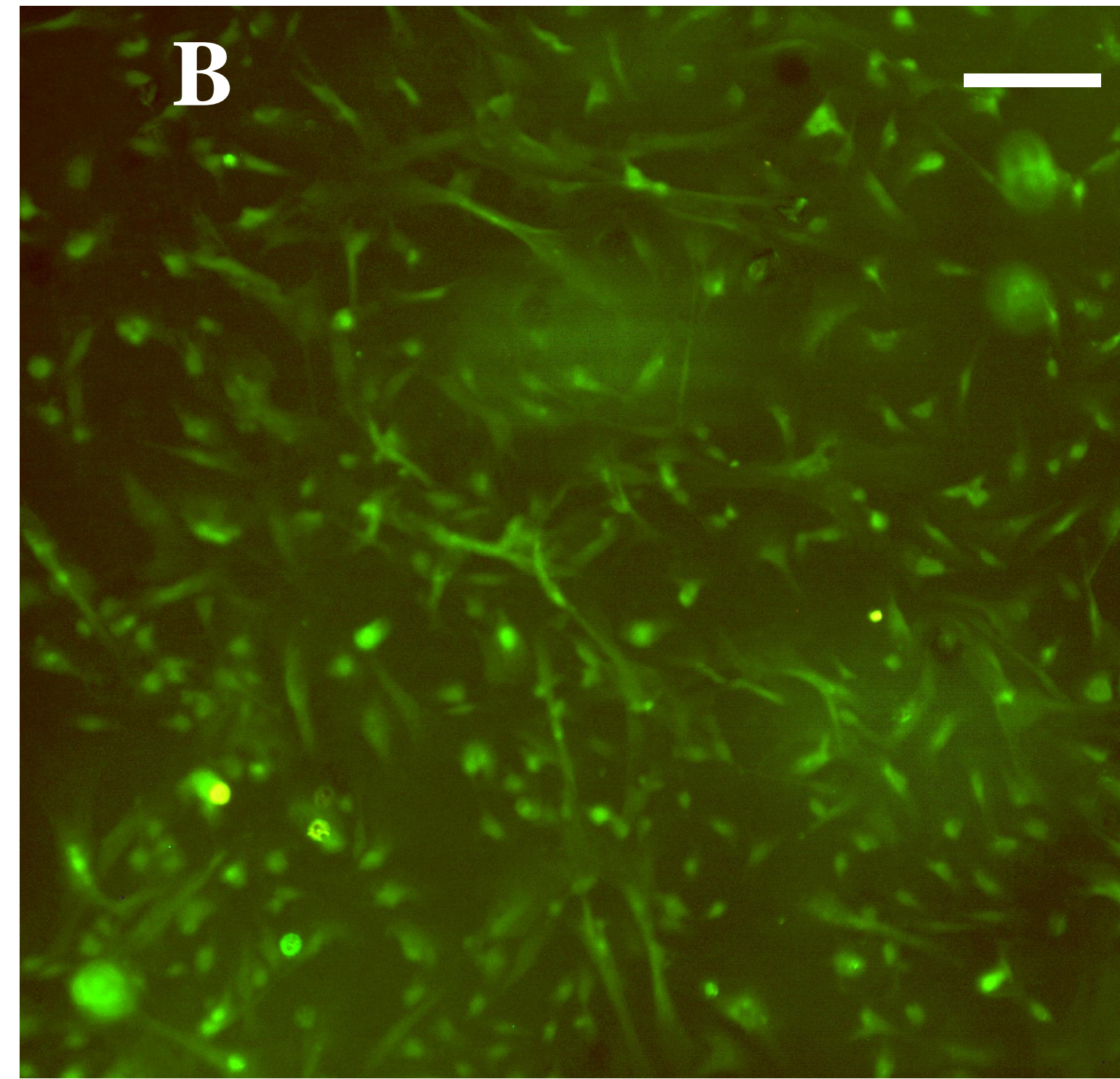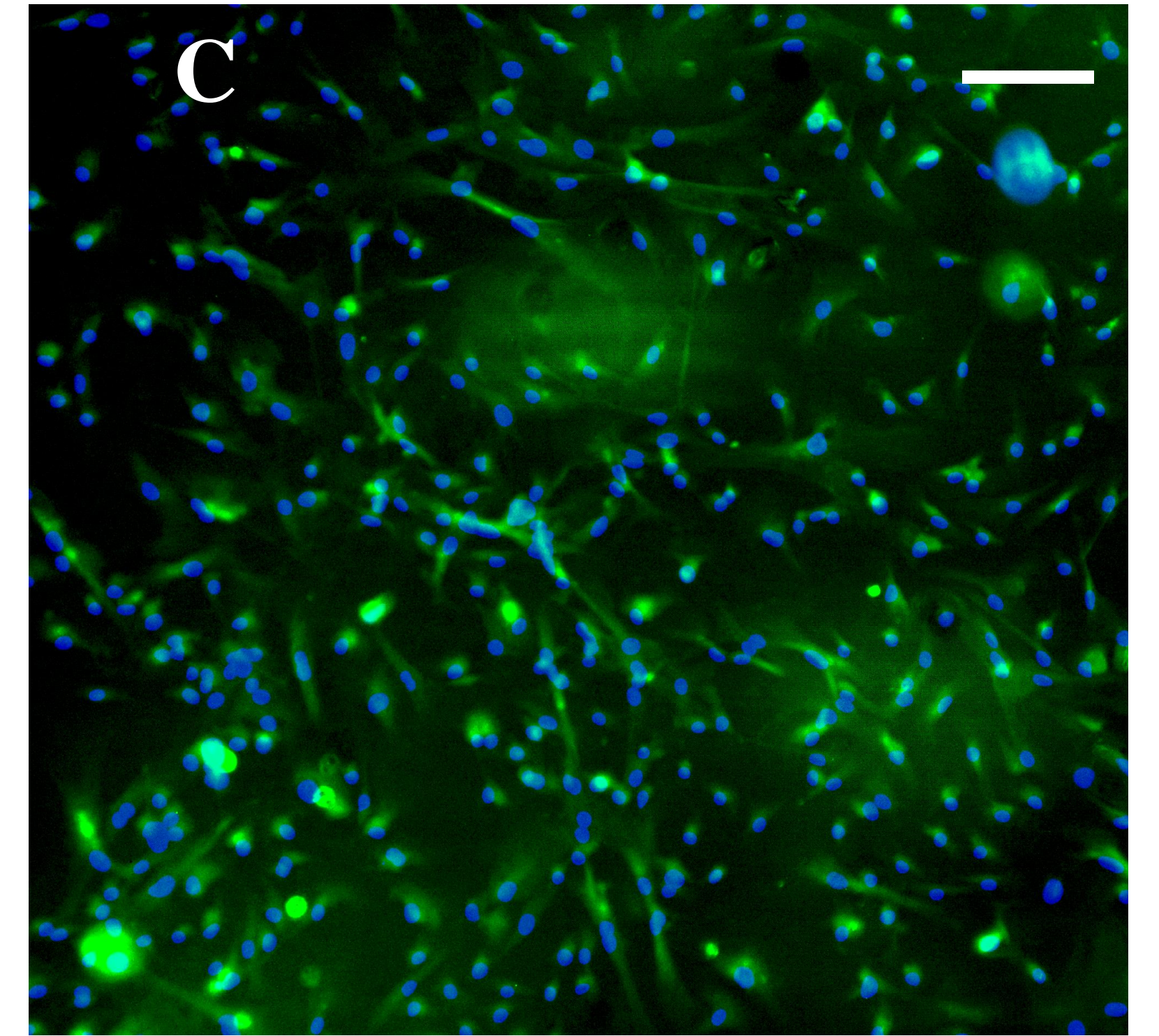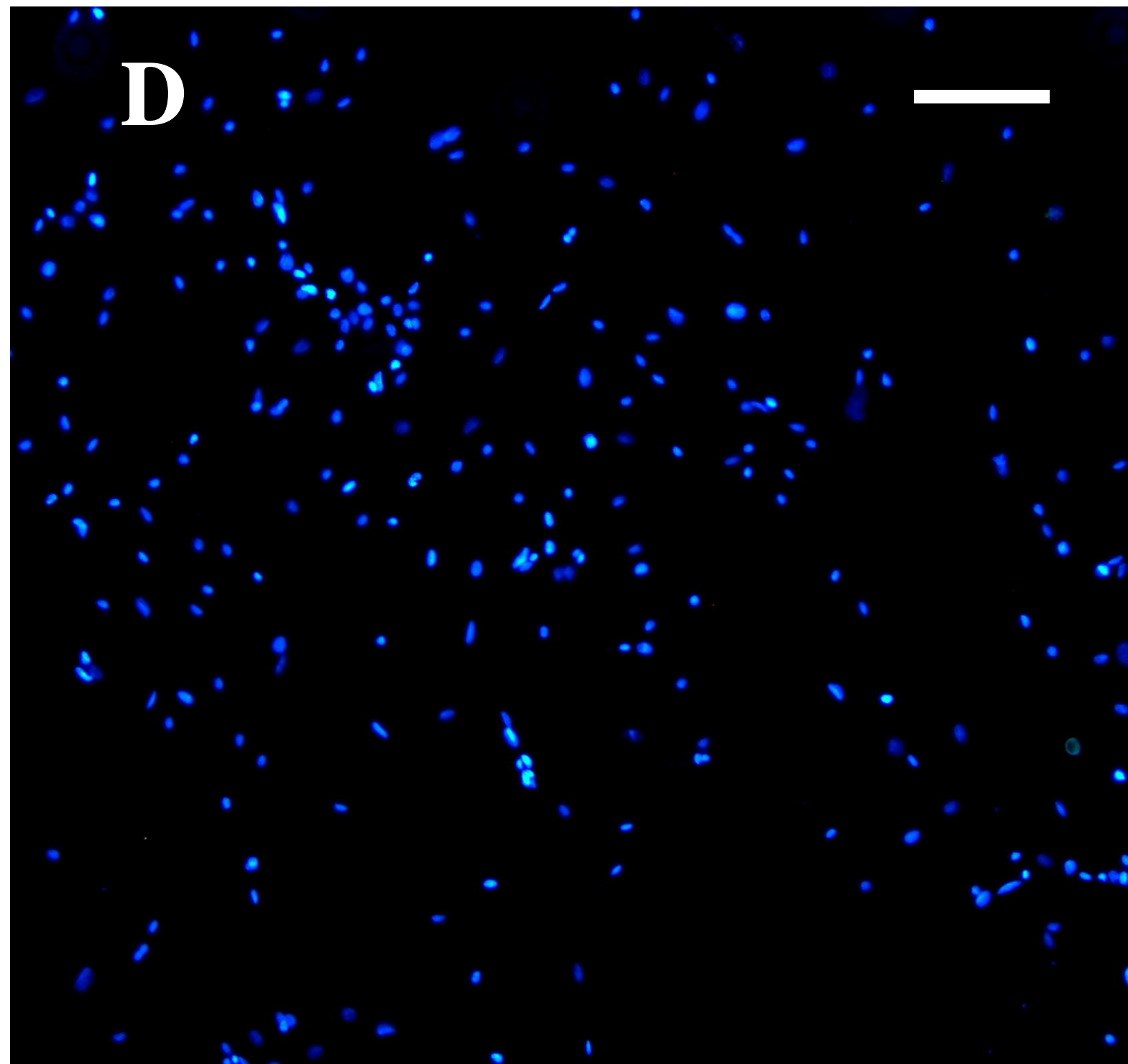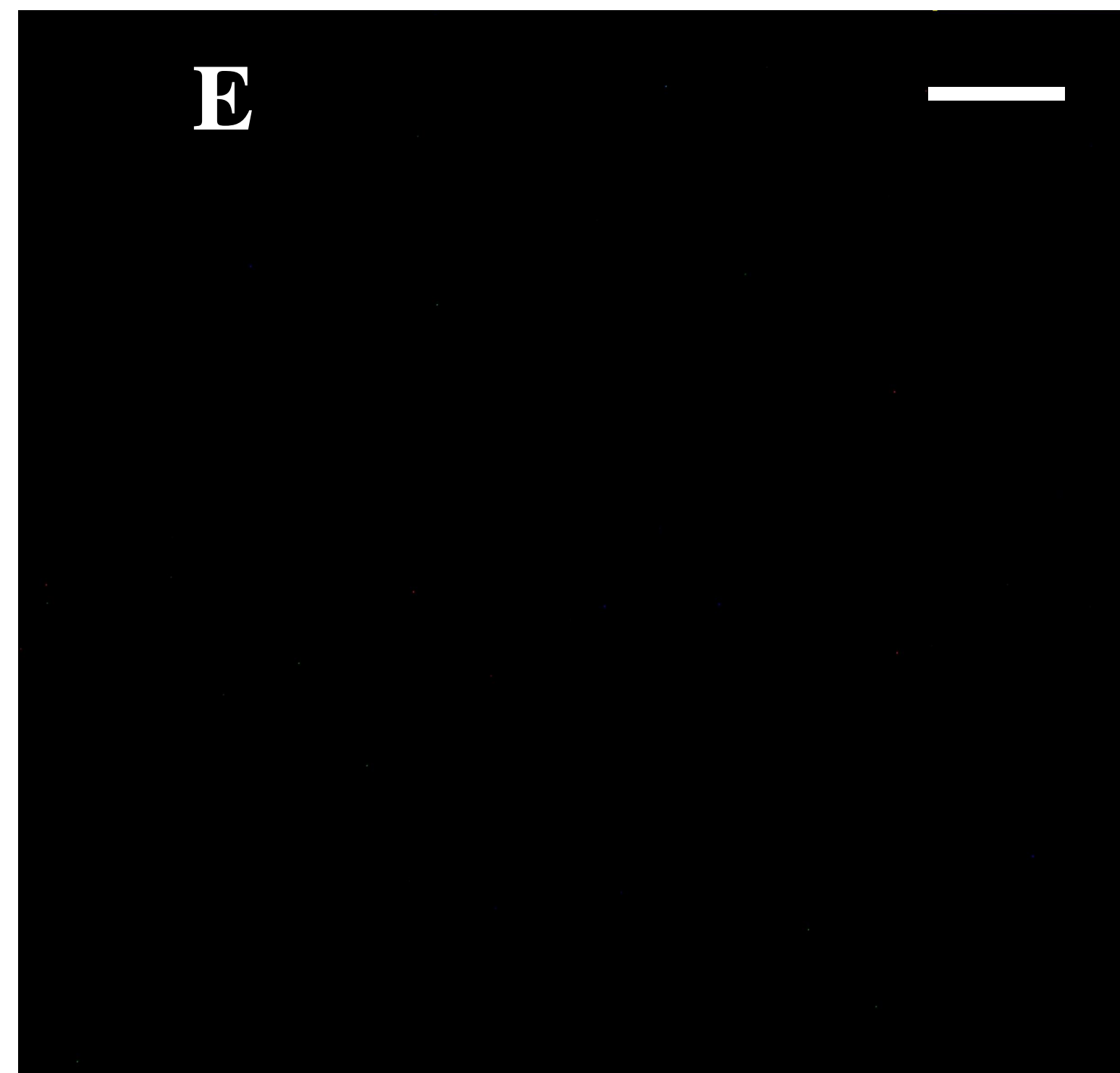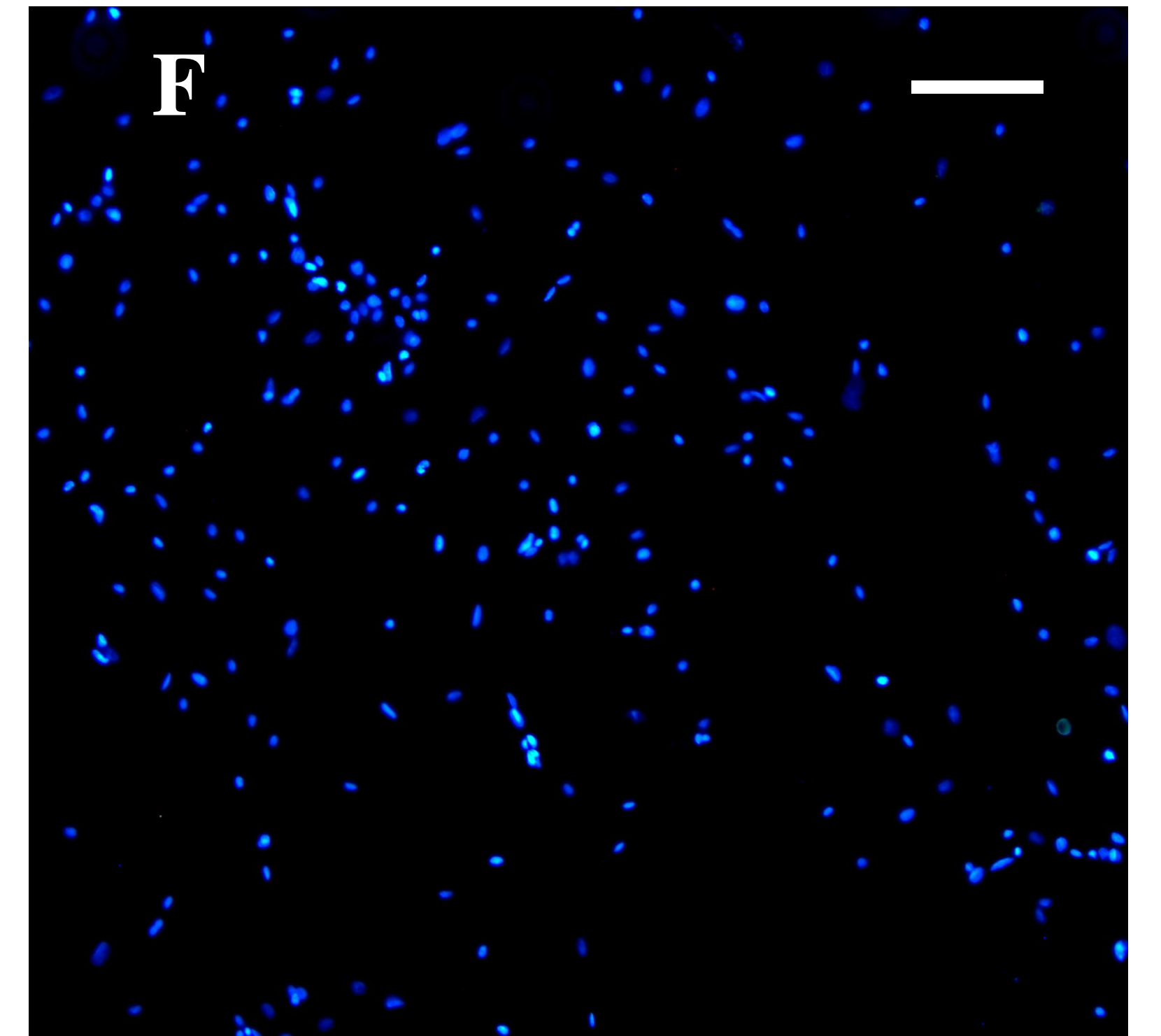

Supplement: Supplementary Figure 2 — Immunofluorescence staining shows the positive expression of vimentin expression (green) and negative expression of cytokeratin-18 (red) in bESCs (Scale bars = 50 μm, 100×). (A) Nuclear staining with DAPI (blue) of bESCs. (B) Immunostaining images of vimentin (green) of bESCs. (C) Immunofluorescence of bESCs. (D) Nuclear staining with DAPI (blue) of bESCs. (E) Immunostaining images of keratin-18 (red) of bESCs. (F) Immunofluorescence of bESCs. [file Data_Sheet_2.PDF]

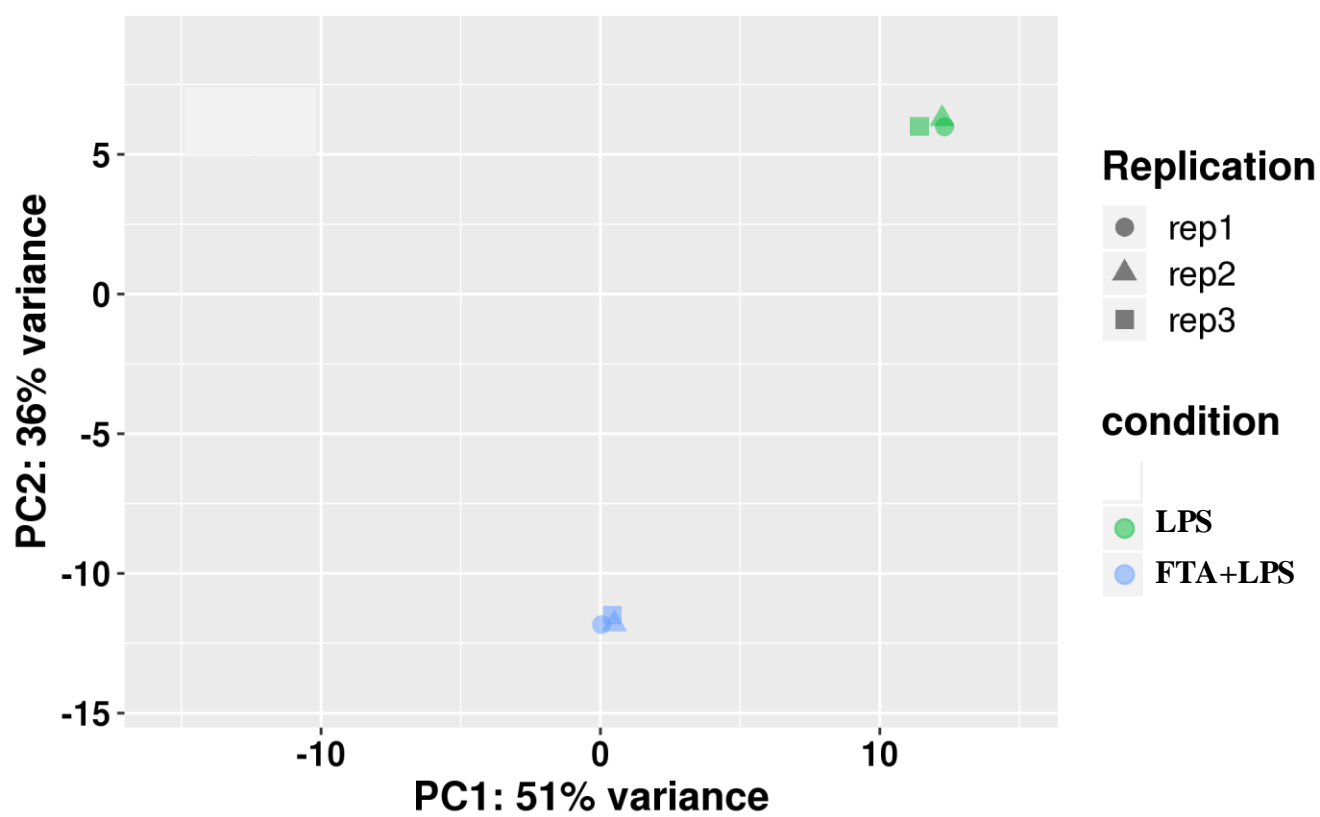

Supplement: Supplementary Figure 4 — Principal Component Analysis (PCA) of transcriptomes of all samples. [file Data_Sheet_4.PDF]
