# Supplementary material for: Role of MicroRNAs in Protective Effects of Forsythoside A Against Lipopolysaccharide-Induced Inflammation in Bovine Endometrial Stromal Cells
Source: Front Vet Sci. 2021 Feb 24;8:642913. doi: 10.3389/fvets.2021.642913 (PMC7943879; doi:10.3389/fvets.2021.642913)
Supplement: Supplementary Figure 3 — The RIN values of RNA including LPS 1, LPS 2, LPS 3, LPS+FTA 1, LPS+FTA 2, LPS+FTA 3. (A) The RIN values of RNA in LPS1 group. (B) The RIN values of RNA in LPS2 group. (C) The RIN values of RNA in LPS3 group. (D) The RIN values of RNA in LPS+FTA1 group. (E) The RIN values of RNA in LPS+FTA2 group. (F) The RIN values of RNA in LPS+FTA3 group. [file Data_Sheet_3.PDF]

Assay Class: Eukaryote Total RNA Nano Created: 2019-08-06 4:40:22 PM
Data Path: C:\...Eukaryote Total RNA Nano_DEDAE00452_2019-08-06_16-40-22.xad Modified: 2019-08-06 5:04:15 PM

Electropherogram Summary Continued ...

LPS11

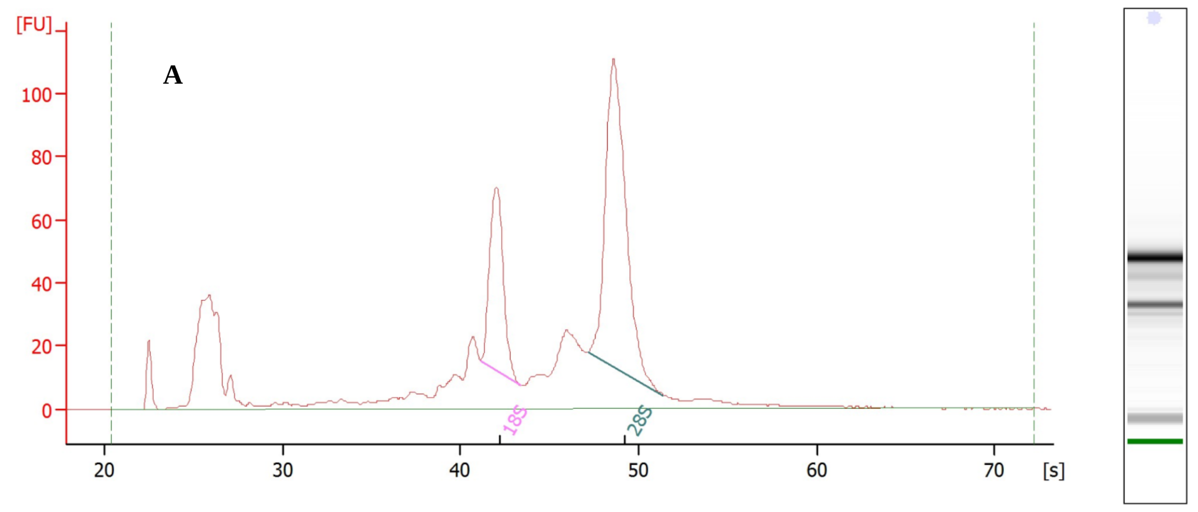

Overall Results for sample 4 : LPS11

RNA Area:	1,078.4	RNA Integrity Number (RIN):	8.7 (B.02.09)
RNA Concentration:	374 ng/µl	Result Flagging Color:	
rRNA Ratio [28s / 18s]:	2.1	Result Flagging Label:	RIN: 8.70

Fragment table for sample 4 : LPS11

Name	Start Time [s]	End Time [s]	Area	% of total Area
18S	41.13	43.38	123.5	11.5
28S	47.20	51.38	264.5	24.5

Assay Class: Eukaryote Total RNA Nano Created: 2019-08-06 4:40:22 PM
Data Path: C:\...Eukaryote Total RNA Nano_DEDAE00452_2019-08-06_16-40-22.xad Modified: 2019-08-06 5:04:15 PM

Electropherogram Summary Continued ...

LPS12

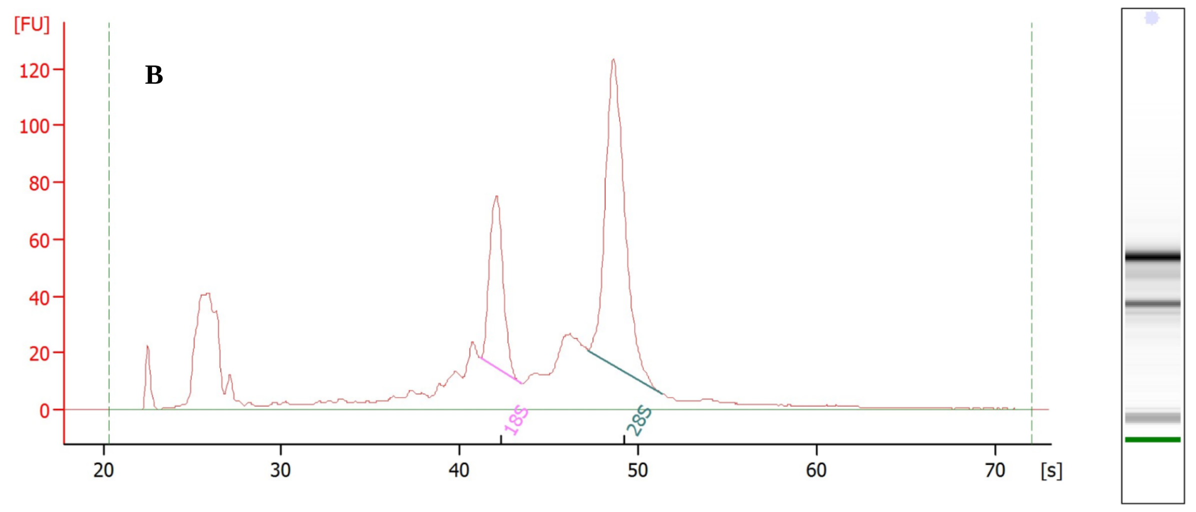

Overall Results for sample 5 : LPS12

RNA Area:	1,204.7	RNA Integrity Number (RIN):	8.5 (B.02.09)
RNA Concentration:	418 ng/µl	Result Flagging Color:	
rRNA Ratio [28s / 18s]:	2.2	Result Flagging Label:	RIN: 8.50

Fragment table for sample 5 : LPS12

Name	Start Time [s]	End Time [s]	Area	% of total Area
18S	41.14	43.43	128.6	10.7
28S	47.19	51.32	279.2	23.2

Assay Class: Eukaryote Total RNA Nano Created: 2019/11/7 14:16:47
Data Path: C:\...Eukaryote Total RNA Nano_DEDAE00182_2019-11-07_14-16-48.xad Modified: 2019/11/7 14:53:06

Electropherogram Summary Continued ...

FTA3

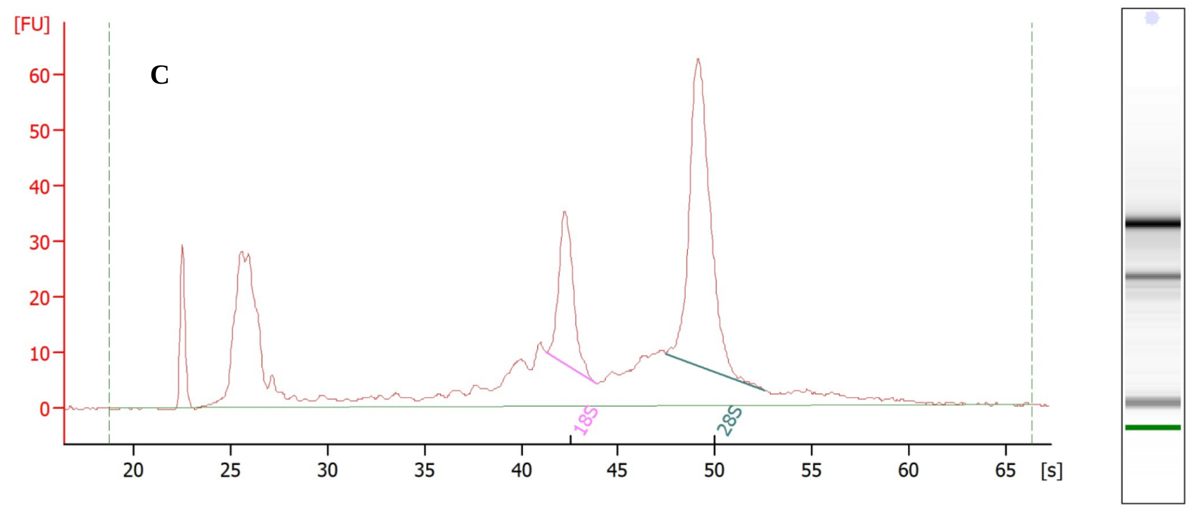

Overall Results for sample 5 : FTA3

RNA Area:	662.2	RNA Integrity Number (RIN):	8.3 (B.02.09)
RNA Concentration:	242 ng/µl	Result Flagging Color:	
rRNA Ratio [28s / 18s]:	2.5	Result Flagging Label:	RIN: 8.30

Fragment table for sample 5 : FTA3

Name	Start Time [s]	End Time [s]	Area	% of total Area
18S	41.30	43.89	56.8	8.6
28S	47.45	52.55	140.7	21.3

Assay Class: Eukaryote Total RNA Nano Created: 2019/11/7 14:16:47
Data Path: C:\...Eukaryote Total RNA Nano_DEDAE00182_2019-11-07_14-16-48.xad Modified: 2019/11/7 14:53:06

Electropherogram Summary Continued ...

FTA1

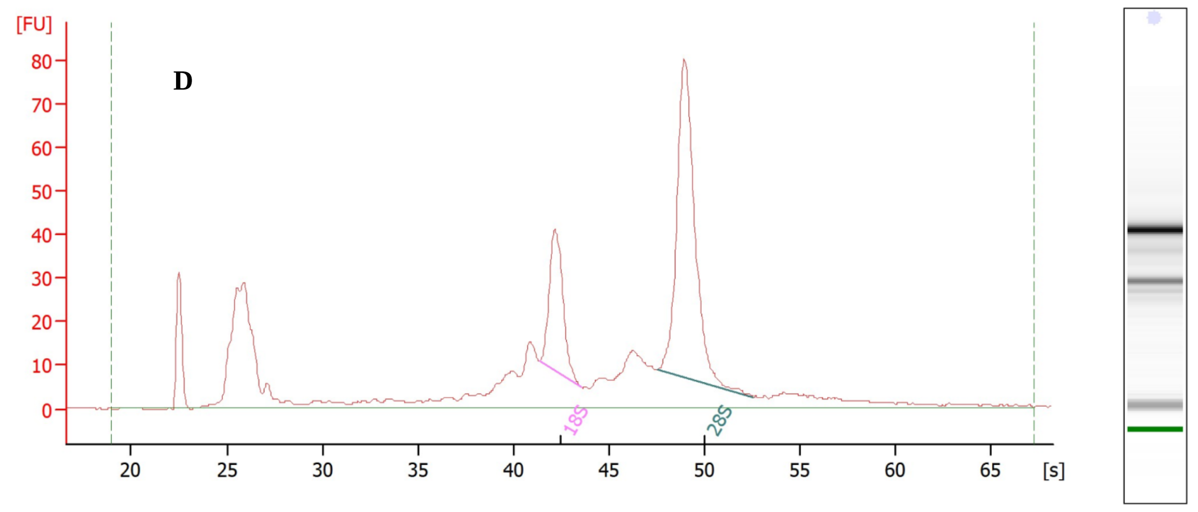

Overall Results for sample 3 : FTA1

RNA Area:	698.3	RNA Integrity Number (RIN):	8.5 (B.02.09)
RNA Concentration:	255 ng/µl	Result Flagging Color:	
rRNA Ratio [28s / 18s]:	2.5	Result Flagging Label:	RIN: 8.50

Fragment table for sample 3 : FTA1

Name	Start Time [s]	End Time [s]	Area	% of total Area
18S	41.35	43.59	64.3	9.2
28S	47.53	52.55	160.9	23.0

Assay Class: Eukaryote Total RNA Nano Created: 2019/11/7 14:16:47
Data Path: C:\...Eukaryote Total RNA Nano_DEDAE00182_2019-11-07_14-16-48.xad Modified: 2019/11/7 14:53:06

Electropherogram Summary Continued ...

FTA2

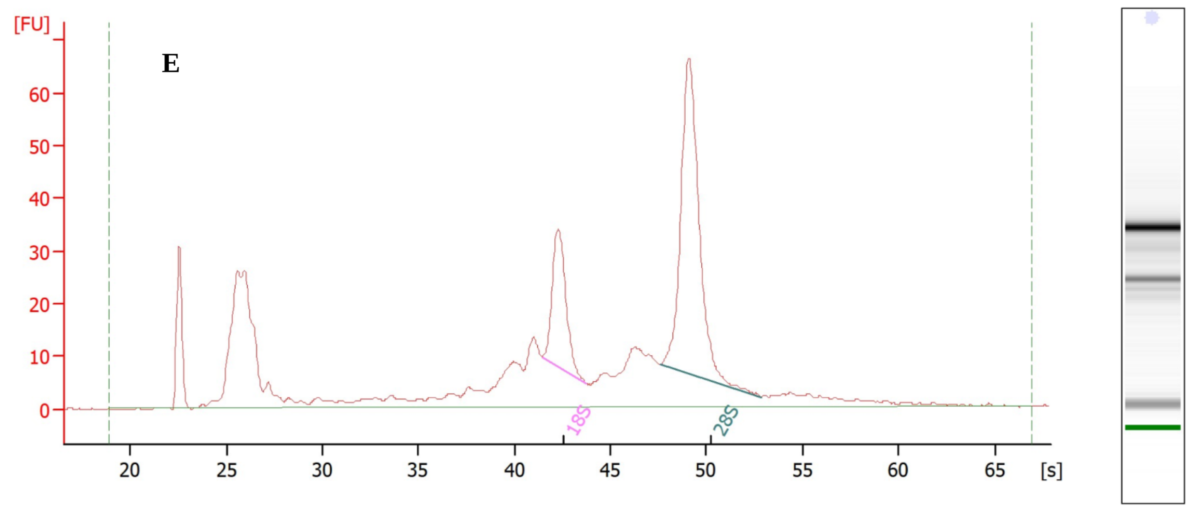

Overall Results for sample 4 : FTA2

RNA Area:	624.1	RNA Integrity Number (RIN):	8.2 (B.02.09)
RNA Concentration:	228 ng/µl	Result Flagging Color:	
rRNA Ratio [28s / 18s]:	2.6	Result Flagging Label:	RIN: 8.20

Fragment table for sample 4 : FTA2

Name	Start Time [s]	End Time [s]	Area	% of total Area
18S	41.46	43.74	50.9	8.2
28S	47.62	52.81	133.1	21.3

Assay Class: Eukaryote Total RNA Nano Created: 2019-08-06 4:40:22 PM
Data Path: C:\...Eukaryote Total RNA Nano_DEDAE00452_2019-08-06_16-40-22.xad Modified: 2019-08-06 5:04:15 PM

Electropherogram Summary Continued ...

LPS13

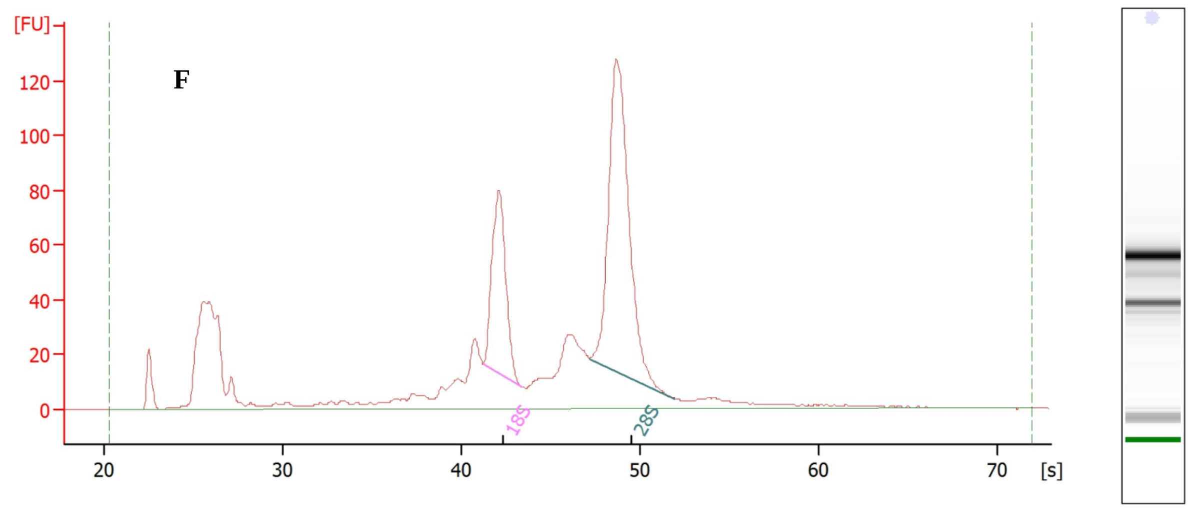

Overall Results for sample 6 : LPS13

RNA Area:	1,195.0	RNA Integrity Number (RIN):	8.8 (B.02.09)
RNA Concentration:	414 ng/µl	Result Flagging Color:	
rRNA Ratio [28s / 18s]:	2.2	Result Flagging Label:	RIN: 8.80

Fragment table for sample 6 : LPS13

Name	Start Time [s]	End Time [s]	Area	% of total Area
18S	41.20	43.33	142.5	11.9
28S	47.14	51.93	306.6	25.7
